# Evaluation of the relationship between the collapsed mechanism and excavation method in tunnels excavated in schists

**DOI:** 10.1038/s41598-022-25767-1

**Published:** 2022-12-08

**Authors:** Ebu Bekir Aygar

**Affiliations:** Fugro Sial Geosciences Consulting Engineering Ltd., 06690 Cankaya, Ankara Turkey

**Keywords:** Solid Earth sciences, Engineering

## Abstract

The problems experienced in tunnels excavated under high cover in graphitic schists generally vary according to the squeezing mechanism. During the tunnel excavation, slips occur on the slickenside surfaces in the tunnel face from time to time and collapse occurs. Most of the time, failures in the support systems are observed due to the squeezing mechanism in the long term in the sections whose tunnel excavations have been completed. In addition, if tunnel excavations are carried out from both entrance faces, it is possible to encounter excessive deformations at the junction points of the tunnels. Especially on weak ground, the importance of the distances between the stages increases in the case of opening the tunnels in the form of top heading, bench, and invert gradually. As a tunnel excavation method, excavation in stages directly affects the stability of the tunnel. Within the scope of this study, the collapsed mechanism in the junction area of the tunnels of the T6 tunnel is examined. For this purpose, 3-dimensional numerical analysis are performed with the Flac3d program. Analysis results are compared with site deformation measurements. As a result, an excavation methodology is proposed for the junction area of tunnels in weak ground.

## Introduction

Problems experienced in tunnel excavation in schists generally occur due to face sliding occurring in the tunnel face and failures in support systems due to squeezing in the excavated parts of the tunnel. When the problems that occur during the tunnel excavation are examined, it is seen that the face slips occur under the shallow cover, while both the face slides and the squeezing mechanism are effective under the high cover^1^. The compression mechanism is investigated in studies conducted on such weak grounds^2–5^. In these studies, it is essential to provide tunnel face and ceiling stability and active and passive support system approaches in order to prevent squeezing have been opened to discussion. In these approaches, Shubert^6^ suggested deformation gaps in support systems, while Hoek^7,8^ suggested that the TH type should be chosen as the sliding type of snap-on steel rib in tunnels. New Austrian Tunneling Method (NATM) in the design of the support system Rabcewicz^9–11^, Rabcewicz and Golser^12^, and Muller^13^ also proposed for squeezing ground. The basic principle in the NATM method is based on the principle of maximizing the bearing capacity of the soil by allowing deformations with a flexible outer arch principle. However, it has emerged that revisions are needed during the support design process according to the principle of a flexible outer arch, which is the basic philosophy of this method^14–16^. Aygar^16^ insisted that, in weak grounds and large-diameter tunnels, the need for rigid lining has emerged instead of the flexible outer belt principle. According to Kontogianni et al.^17^ stated that 50% of the deformations that occur are due to the time-dependent creep effect and face propagation. For squeezing ground, Jethwa^2^ emphasized that the support system pressure should be 2–3 times higher than the short-term support system pressure in his study in the Chhibro tunnel in the Himalayas. Malan and Basson^18^ stated that the squeezing mechanism increases with increasing depth and decreasing rock mass properties. Sing et al.^19^ defined the squeezing mechanism according to the NGI (Nowagin Geological Institute) system (Q). Hoek and Marinos^3^ and Jethwa et al.^2^ classified the compression mechanism according to the compressive strength of the rock mass and the in-situ stresses. Goel et al.^20^ determined the squeezing conditions according to the N (Rock Mass Number) coefficient and showed it graphically. According to Aydan et al.^21^, on the other hand, divided the compaction into 5 different categories and classified them according to the very squeezing ground from the nonsqueezing conditions.

As a common view in all studies^2,3,20–24^, they determined that the ceiling stability and face stability are critical in the tunnel face. Barla^25^ stated that compression is very important in the long term and that the support system design should be performed accordingly. On the other hand, Chern et al.^26^ also stated that stability problems are likely to be encountered when the strain exceeds 1%. Shrestha and Panthi^27^ analyzed plastic deformations in schist and mica ganys. Panthi^28^ indicates that plastic deformations in weak rocks occur when tangential stresses exceed rock strength. The tunnel excavation method is another important factor affecting tunnel design in compacted soils. Here, discussions continue on whether the excavation method in large-diameter tunnels is the classical tunneling method, gradual excavation (top heading, bench and invert), or the full section tunneling method^29–33^. In addition, support design principles are classified as active and passive approaches. What kind of support system design will be carried out in compacted soils is also a matter of discussion^1,6,25^. Aygar^16^ suggested the implementation of an active support system instead of a passive approach in large-diameter tunnels opened in squeezing ground and stated that the NATM principles should be revised.

As can be seen, the design of the support system is very important in tunnels exposed to squeezing on weak soils. Within the scope of this study, the problems and collapse section of the T6 tunnel excavated in the schists and under high overburden within the scope of the Bursa Yenişehir High Speed Railway Project is evaluated. It is explained whether the main cause of the collapse is due to the support system or the excavation methodology. Excavation methodology in squeezing ground is proposed and limitations is shown. For this purpose, 3D analyzes were performed with the Flac3d program, and the tunnel collapse section is modeled exactly according to the construction phase. By examining the deformations that occurred, the main cause of the collapse is revealed.

## Tunnel specifications

**Figure 1:**
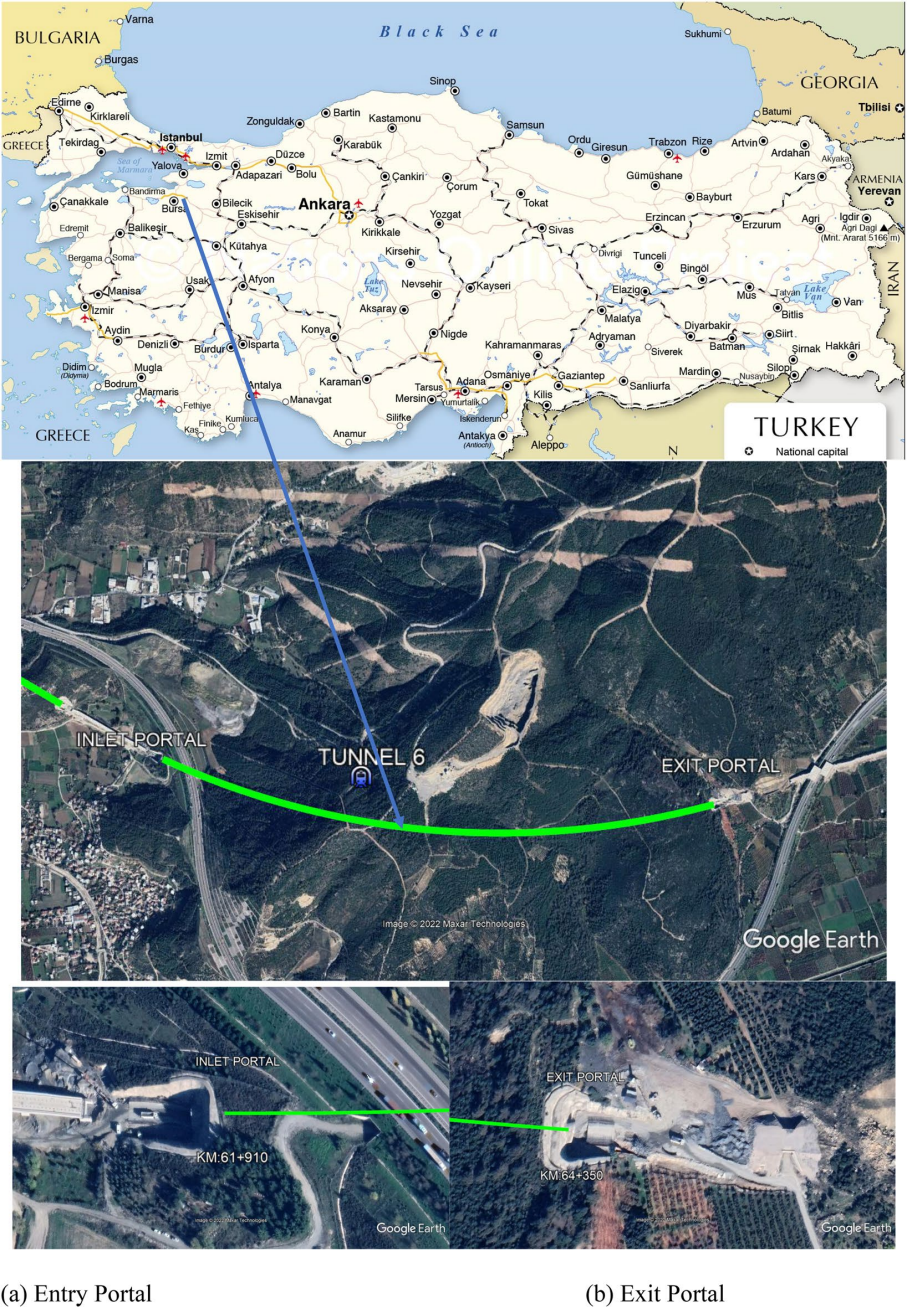
Location map of the T6 tunnel (National Online Project, https://www.nationsonline.org/oneworld/map/turkey-map.htm) and its entry and exit portals on Google Earth view (the Google earth pro 7.3.4.6442 (64bit).

The T6 tunnel is between km: 61 + 910.00 and Km: 64 + 350.00 within the scope of the Bursa Yenişehir High Speed Train Project, and its total length is 2440 m (Fig. 1). The tunnel overburden height is around 200 m maximum^34^. During the tunnel excavation, serious deformations were encountered between km: 62 + 822.00 and km: 63 + 026.00. Reprofiling and strengthening works were carried out continuously in these sections. The excavations in the top heading of the tunnel were completed, but the bench and invert excavations were not carried out in the 28 m section between km: 62 + 910 and km: 62 + 938. In this process, the deformations in the tunnel continued to increase at the junction points, and a collapse occurred at the junction of the tunnel and the tunnel was closed.

**Figure 2:**
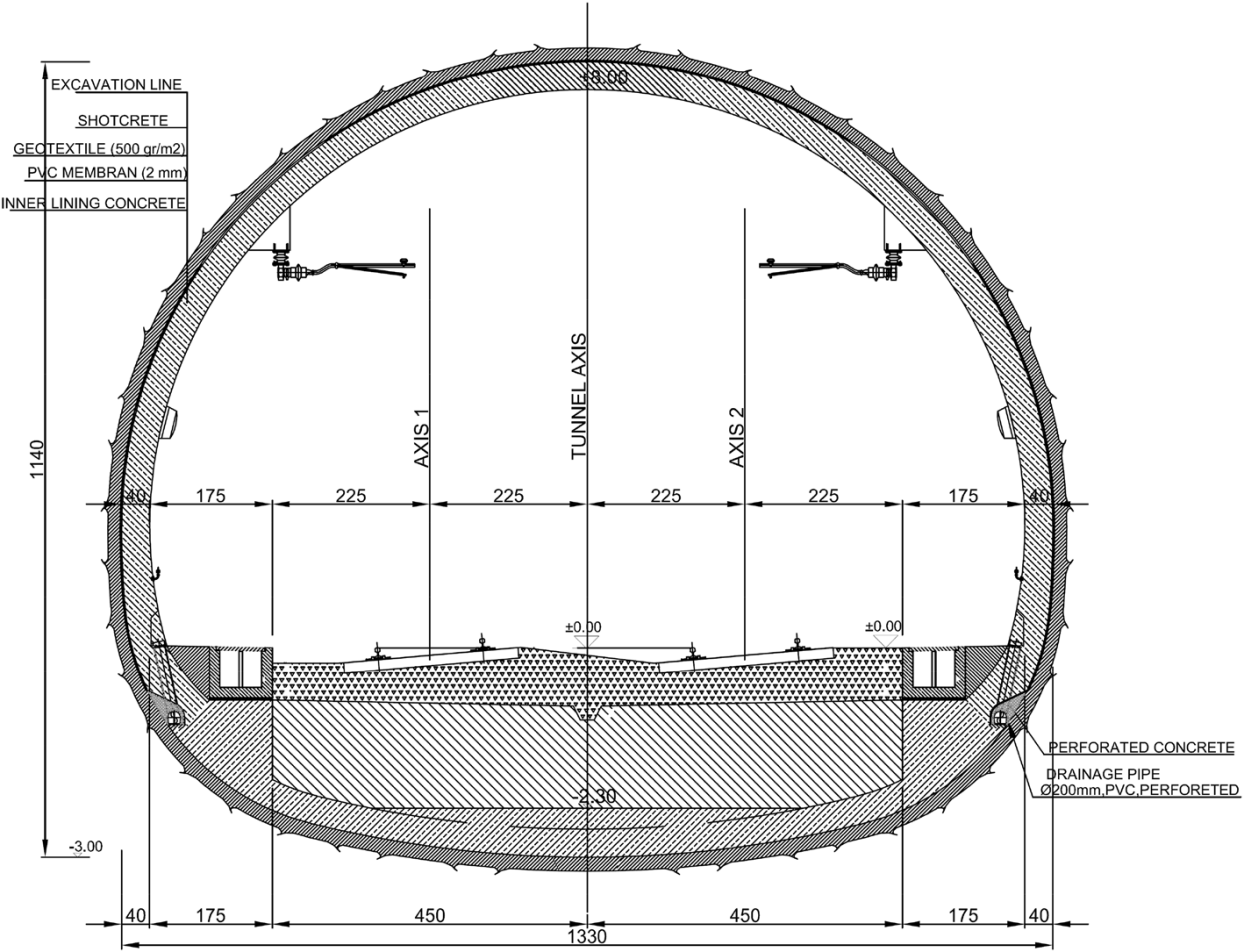
Typical cross-section of the T6 tunnel.

The Google Earth image of the tunnel route is given in Fig. 1. The tunnel is designed as a single tube, its height is 8.0 m and the excavation diameter is 13.5 m (Fig. 2). The T6 tunnel was designed according to the principles of the New Austrian Tunneling Method^9–13^.

### Geological and geotechnical conditions in T6 tunnel

**Figure 3:**
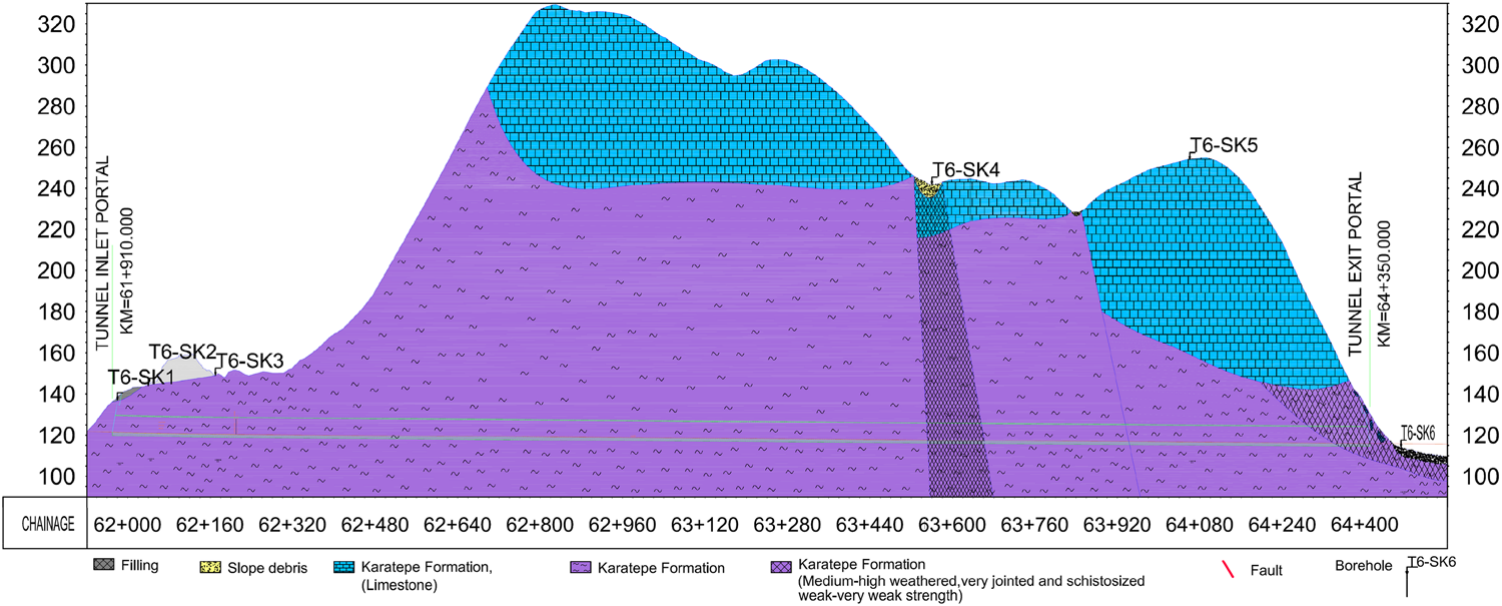
T6 tunnel geological and geotechnical profile^34^.

Triassic Karatepe formation is encountered along the route. Considering the boreholes and geological mapping data, the section along the route passes through the Karatepe metasandstone-schists member and limestone unit, which consists of alternations of sandstone, metasandstone, shale, mudstone, metaconglomerate, limestone, tuff, agglomerate, and spilitic basalt. The geological profile is given in Fig. 3.

The rock mass parameters determined for the schist unit in the tunnel are given in Table 1.

**Table 1:** Geotechnical parameters.

Deformation module (MPa)	Cohesion c (kPa)	Internal friction angle (ϕ)	Unit Volume Weight (kN/m^3^)	Compressive strength of rock mass (σ_cm_) (MPa)	Poison ratio υ
250	50	30	22	0.17	0.3

### Problems encountered in the tunnel

**Figure 4:**
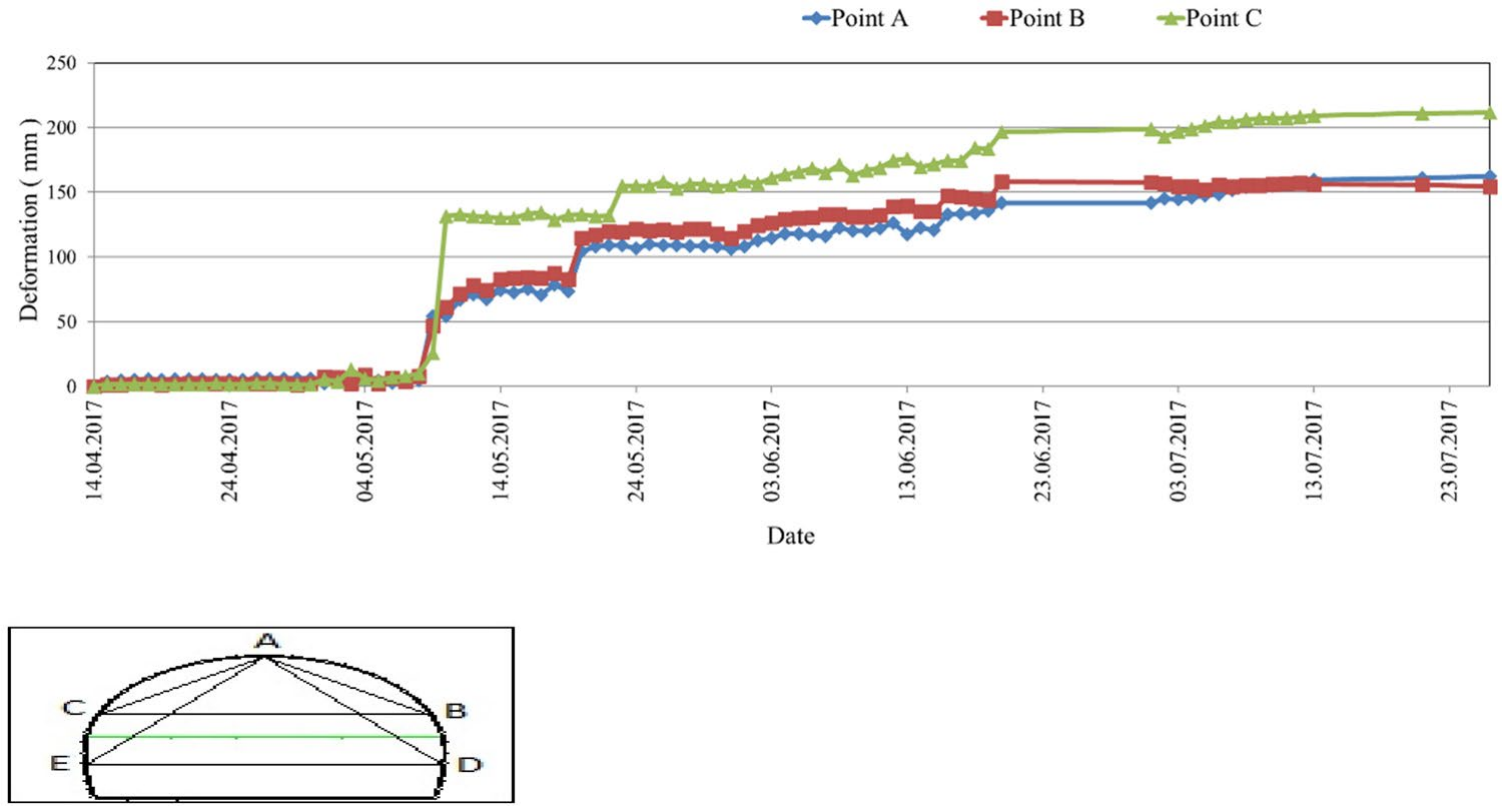
Deformation measurements were taken at km: 62 + 881.

**Figure 5:**
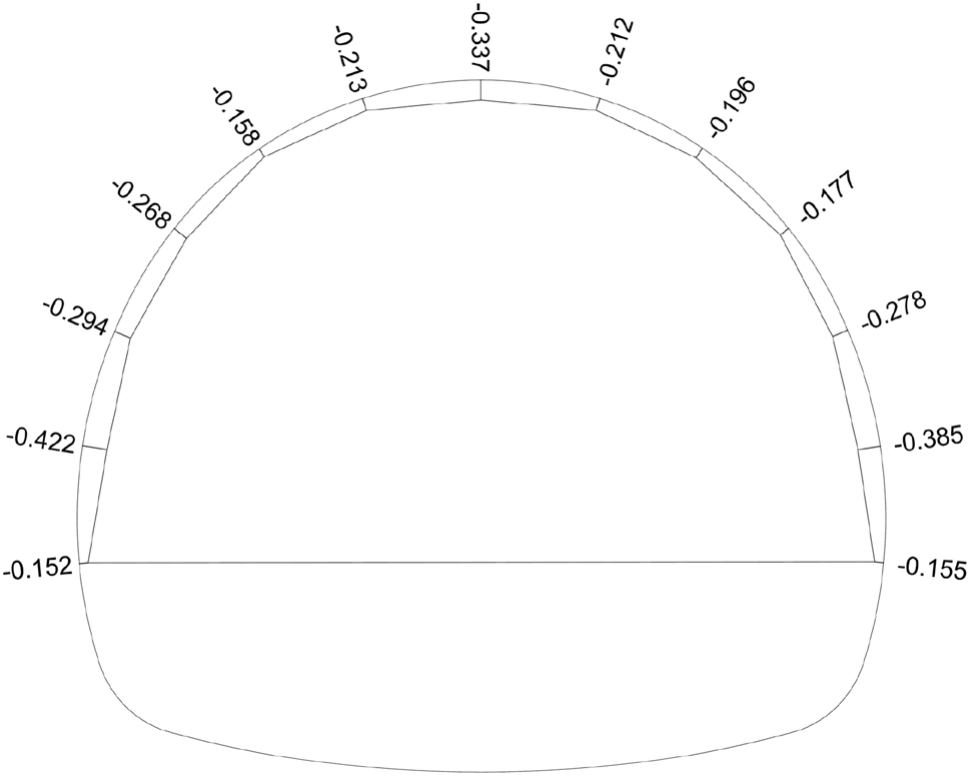
km: 62 + 832 deformation section (in m).

During the tunnel excavation, deformations occurred in the tunnel continuously. As a result of the deformations, cracks in the shotcrete and ruptures in the bolts appeared. A continuous increase in deformations was observed during excavation in the tunnel (Fig. 4), and it was stabilized after the bench and invert excavations. At most points along the tunnel profile, deformations have penetrated the section (Fig. 5). The cross-section measurement is taken after the excavation completed.

**Figure 6:**
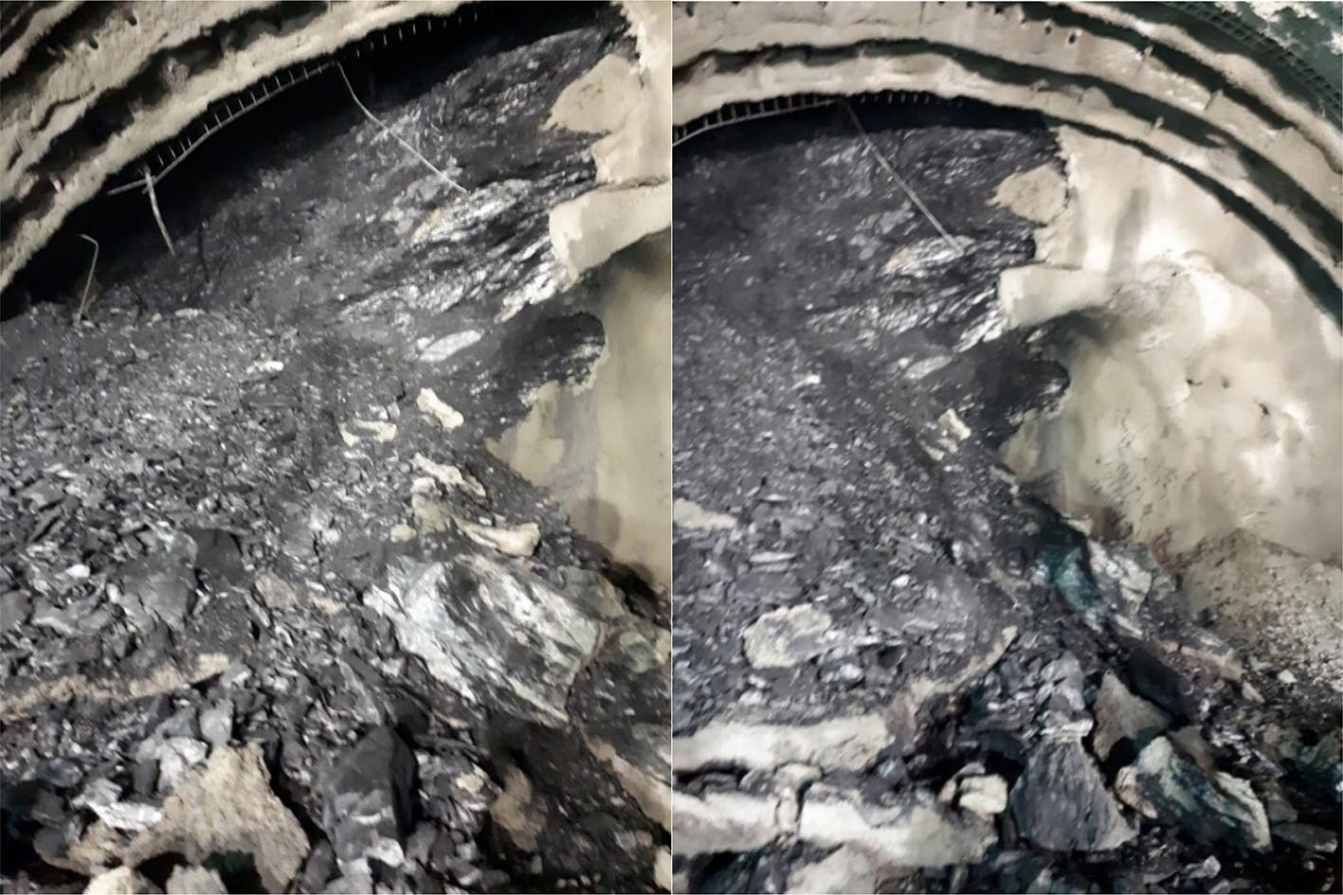
Tunnel face excavation view in the graphitic schist.

One of the biggest problems encountered in the tunnel face was the slides on the slickenside surfaces on the schists surface (Fig. 6). After the excavation, the stability of the face could not be ensured. This situation adversely affected the tunnel support systems. As can be seen from Fig. 6, the most important factor affecting the rock mass parameters is the predominance of slippery surfaces in the schists. This situation both caused problems in the stability of the face during the excavation and caused failures in the support systems due to squeezing in the long term.

**Figure 7:**
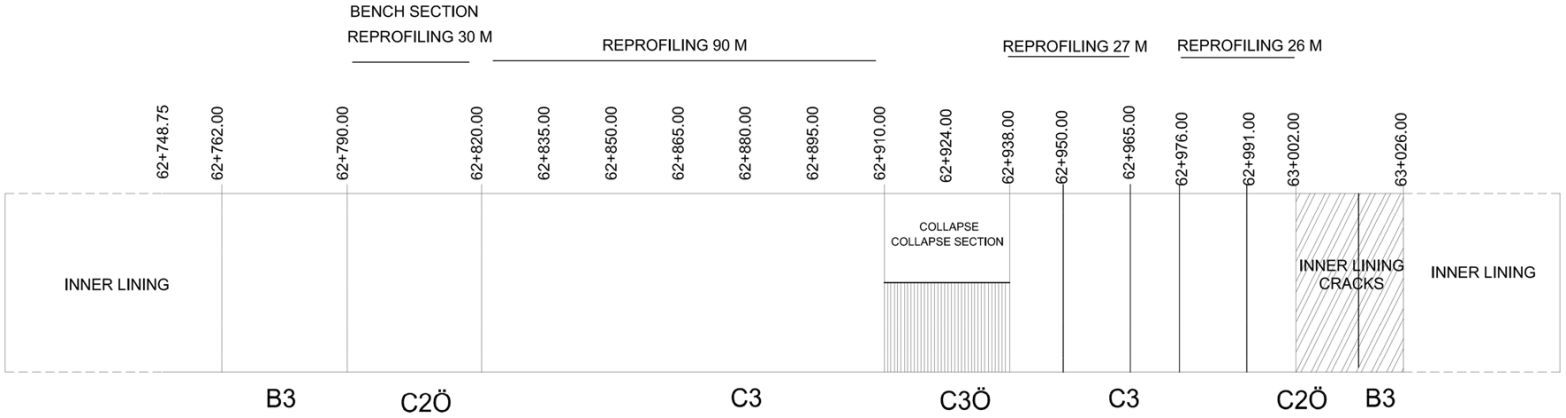
Tunnel excavation situation.

Tunnel excavation continued from both entrances. The tunnel excavation was completed up to km: 62 + 938 from the exit side and the bench excavations were completed up to km: 62 + 910 in the entrance section. The top heading of the 28 m section in between has also been excavated (Fig. 7). In addition, inner lining concrete has been completed from km: 63 + 026 at the tunnel exit to 62 + 762 from the tunnel entrance.

**Figure 8:**
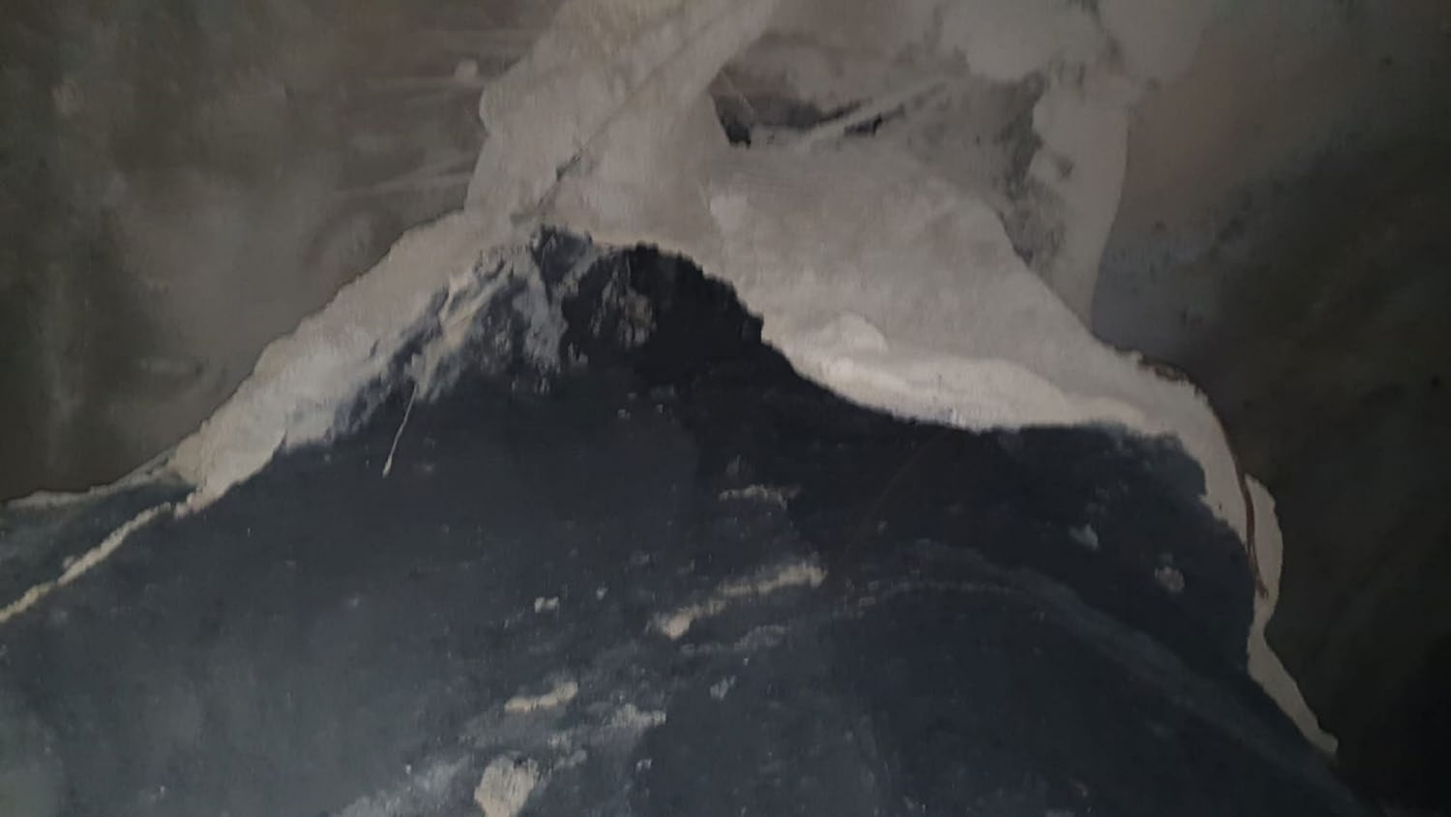
The tunnel collapsed section between km: 62 + 910 to 62 + 938.

After this stage, because of the extreme deformations experienced in the 28 m section that was not excavated, a collapse occurred in the tunnel, and this section of the tunnel was completely closed (Fig. 8).

## Evaluation of tunnel squeezing conditions and tunnel behaviour

One of the biggest problems experienced in tunnels collapsed in schists is that it develops due to squeezing. For this reason, it is imperative to examine the squeezing situation in the design of tunnel support systems. The equations determined according to Sakurai^5^, Jethwa et al.^2^ and Hoek and Marinos^3^ are used to examine the compression situation.

Sakurai^5^ correlated the compressive strength of the rock mass with the strain in the tunnel to define compression.

Sakurai^5^, in his study, examined the squeezing conditions in different underground structures. Equation (1) has been proposed for the sections that require a special support system due to the encountered problems. Here, depending on the compressive strength of the rock mass, the parts under the curve drawn according to the strain do not require a special support system, while the sections above the line require a special support system.

Here, Sakurai^5^ proposed Eq. (1) to determine the strain.

1 $$\upvarepsilon_{{{\text{pc}}}} = {1}.0{73}\sigma_{{{\text{cm}}}}^{{ - 0.{318}}} .$$

The compressive strength (σ_cm_) of the rock mass was 0.17 MPa and the Ɛpc value is calculated as 1.88.

**Figure 9:**
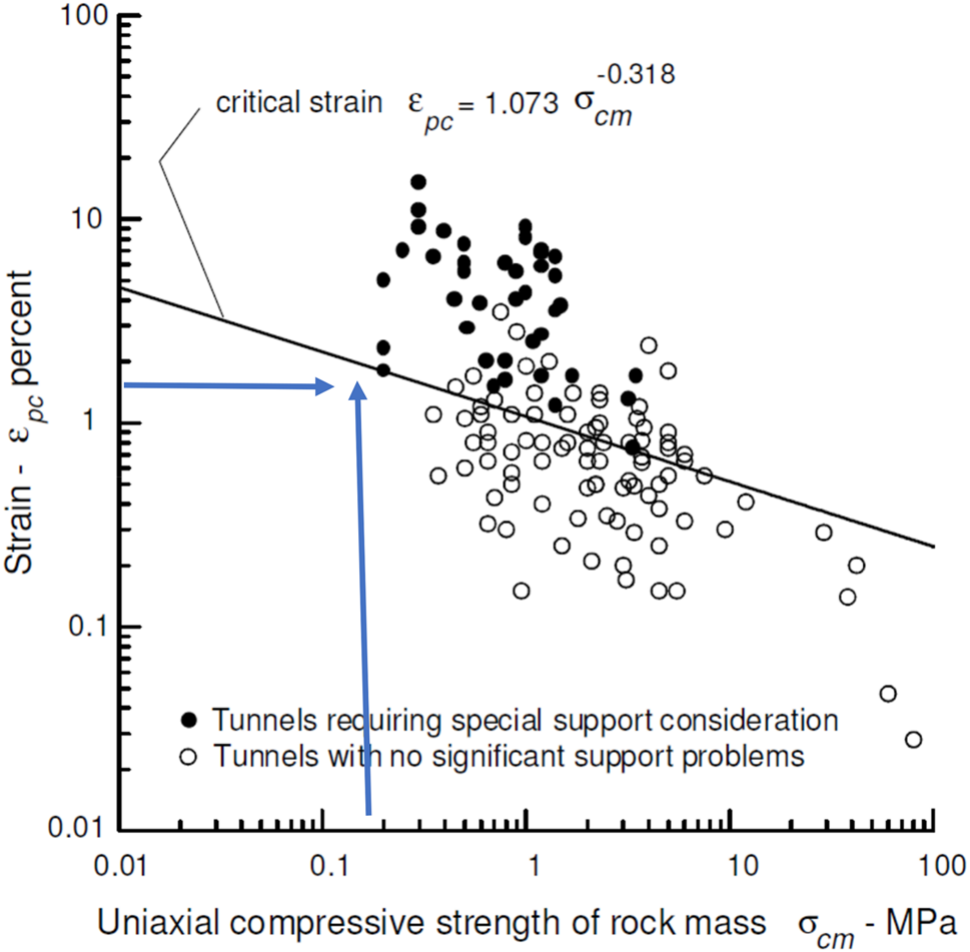
Strain for different rock mass strengths^5^.

In Fig. 9, the squeezing condition is below the critical line.

Jethwa et al.^2^, on the other hand, defined compression as depending on the N_c_ coefficient. N_c_ is given in Eq. (2).

2 $$Nc=\frac{{\sigma }_{\text{cm}}}{Po}=\frac{{\sigma }_{\text{cm}}}{\gamma \times h}.$$

σ_cm_: uniaxial compressive strength of the rock mass, h: height, γ: unit weight.

The N_c_ value is calculated as 0.04. According to the Nc value, it shows a high compression status according to Table 2.

**Table 2:** Squeeing degree according to Jethwa et al.^2^.

Degree of squeezing	Ranges
High	< 0.4
Moderate	0.4–0.8
Slightly	0.8–2
Non-squeezing	> 2

Hoek and Marinos^3^ defined the degree of squeezing as similar to Jethwa et al.^2^, depending on the compressive strength of the rock mass and in-situ stress. The strain value is calculated based on these two values. Strain value (Ɛ) is given in Eq. (3).

3 $$\upvarepsilon = 0.{2} \times (\sigma_{{{\text{cm}}}} /{\text{p}}_{0} )^{{ - {2}}} .$$

**Figure 10:**
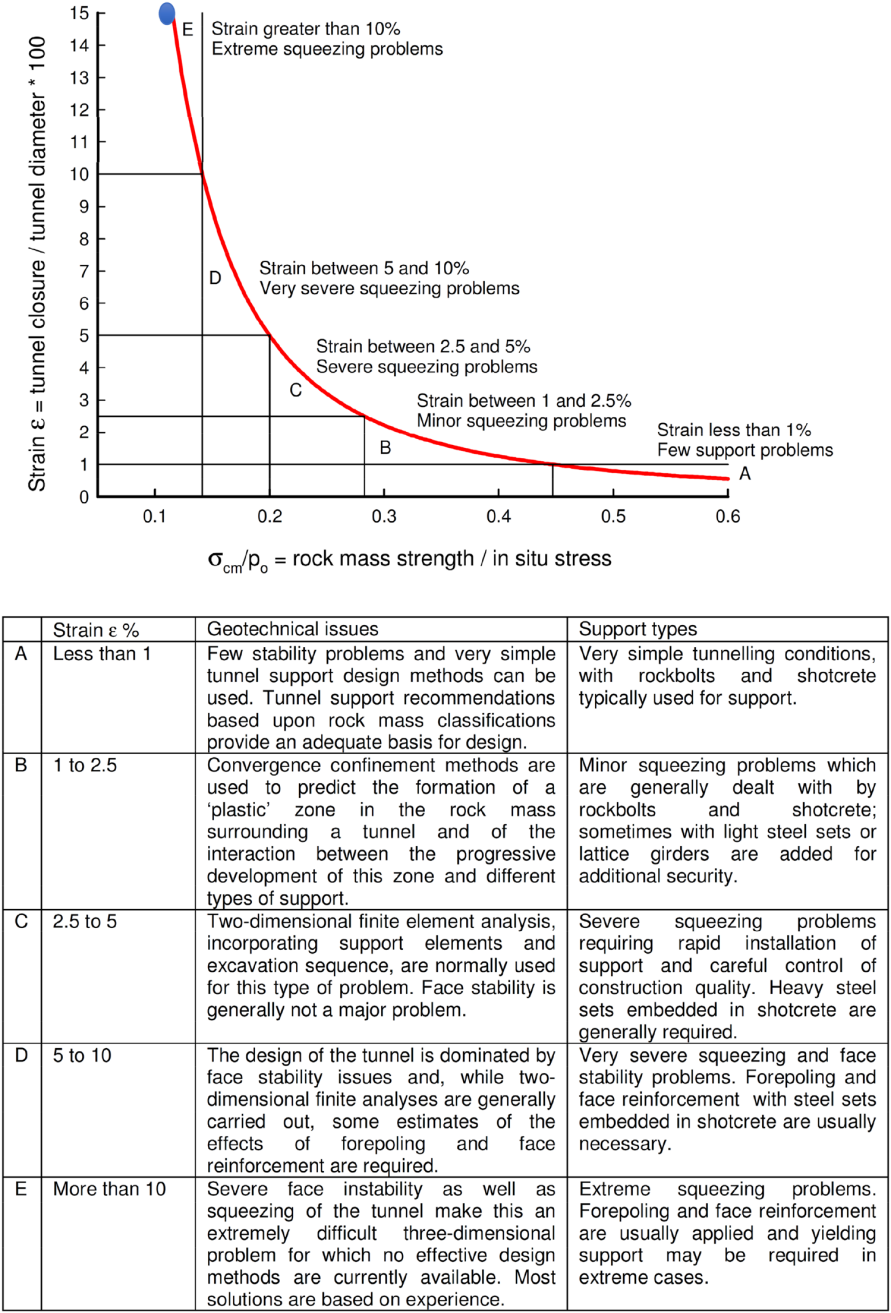
Approximate relationship between strain and the degree of difficulty associated with tunnelling through squeezing rock. Note that this curve is for tunnels with no support^3^.

The ε value is calculated as 133. In Fig. 10, the relationship between strain value and σ_cm_/p_0_ is drawn. Here, the squeezing situation is determined as an extreme squeezing problem.

According to Hoek and Marinos^3^ he stated that there will be serious stability problems in the tunnel and said, “This is an extremely difficult three-dimensional problem for which no effective design methods are currently available. Most solutions are based on experience”.

## Analytical solutions and tunnel support reaction curves

For the design of the support systems according to the squeezing problem in the tunnel, analytical solutions and the reaction curves of the support systems must be determined. Many researchers have carried out studies about this subject^8,35–38^.

It is considered to be homogeneous and under hydrostatic pressure in analytical solutions. The Mohr Coulomb criterion is used as the renewal criterion and the tunnel is assumed to be circular^35^. In Table 3, closed system equations are given.

**Table 3:** Closed form solution equation^8,35^.

Mohr Colulmb Criteria (4)	$\sigma {1}^{^{\prime}}={\sigma }_{\text{cm}}+k\sigma {3}^{^{\prime}}$	Radius of the plastic zone rp when p_i_ = 0 (10)	$$rp=ro[(\frac{2\left(p0\left(k-1\right)+{\sigma }_{\text{cm}}\right)}{\left(1+k\right)\left(\left(k-1\right)pi+{\sigma }_{\text{cm}}\right)}]^\frac{1}{\begin{array}{c}k-1\\ \end{array}}$$
The uniaxial compressive strength of the rock mass σ_cm_ (5)	${\sigma }_{\text{cm}}=\frac{2{c}^{^{\prime}}\text{cos}{\varnothing }^{^{\prime}}}{1-\text{sin}{\varnothing }^{^{\prime}}}$	Inward radial displacement *u*_*ip*_ (11)	$uip=\left(\frac{ro\left(1+\vartheta \right)}{Em}\right)[2\left(1-\vartheta \right)\left(p0-pcr\right){\left(\frac{rp}{r0}\right)}^{2}-\left(1-2\vartheta \right)\left(p0-pi\right)]$
The slope *k* of the versus σ'_1_ − σ '_3_ (7)	$k=\frac{1+\text{sin}{\varnothing }^{^{\prime}}}{1-\text{sin}{\varnothing }^{^{\prime}}}$	Percent strain, Ɛ (12)	$\varepsilon \%=\left(\frac{ui}{ro}\right)\times 100=[0.2-0.25\left(\frac{pi}{p0}\right){\left(\frac{{\sigma }_{\text{cm}}}{p0}\right)}^{2.4\left(\frac{pi}{p0}\right)-2})$
Critical support pressure *p*_*cr*_ (8)	$Pcr=\frac{2p0-{\sigma }_{\text{cm}}}{1+k}$	Radius of plastic zon when pi (13)	$\left(\frac{rp}{r0}\right)=(1.25-0.625\left(\frac{pi}{p0}\right){\left(\frac{{\sigma }_{\text{cm}}}{p0}\right)}^{\left(\frac{pi}{po}\right)-0.57})$
Radial elastic displacement *uie* (9)	$uie=\frac{r0\left(1+\vartheta \right)\left(p0-pi\right)}{Em}$		

*rp* = Plastic zone radius, *ui* = Tunnel sidewall deformation, *ro* = Original tunnel radius in metres, *pi* = Internal support pressure, *po* = In situ stress = depth below surface × unit weight = $p0=\gamma \times h$, *σ*_*cm*_ = Rock mass strength = 2 cosø/(1 − sinø), *Em* = Young’s modulus or deformation modulus and, υ = Poisson’s ratio, σʹ_1_ = the axial stress at which failure occurs, σʹ_3_ = the confining stress, *c*ʹ = the cohesive strength, øʹ = the angle of friction of the rock mass.

The in-situ stress at 200 m overburden height is calculated as p_0_ = 0.022 × 200 = 4.4 MPa. If the compressive strength of the rock mass is σ_cm_, it is 0.17 MPa, and the σ_cm_/P_0_ ratio is 0.04. The displacement at the tunnel face is 41 cm and the plastic displacement around the tunnel is 2.69 m. Table 4 presents the summary table.

**Table 4:** Analytical solution results.

Height h (m)	Rock mass strength σ_cm_ (MPa)	In situ stress P_0_ (MPa)	σ_cm_/P_0_	Plastic zone radius r_p_ (m)	Strain Ɛ (%)	Total deformation u_i_ (m)	Tunnel face deformation u_if_ (m)	Critical support pressure P_cr_ (MPa)
200	0.17	4.4	0.04	33	41	0.53	0.41	2.16

**Figure 11:**
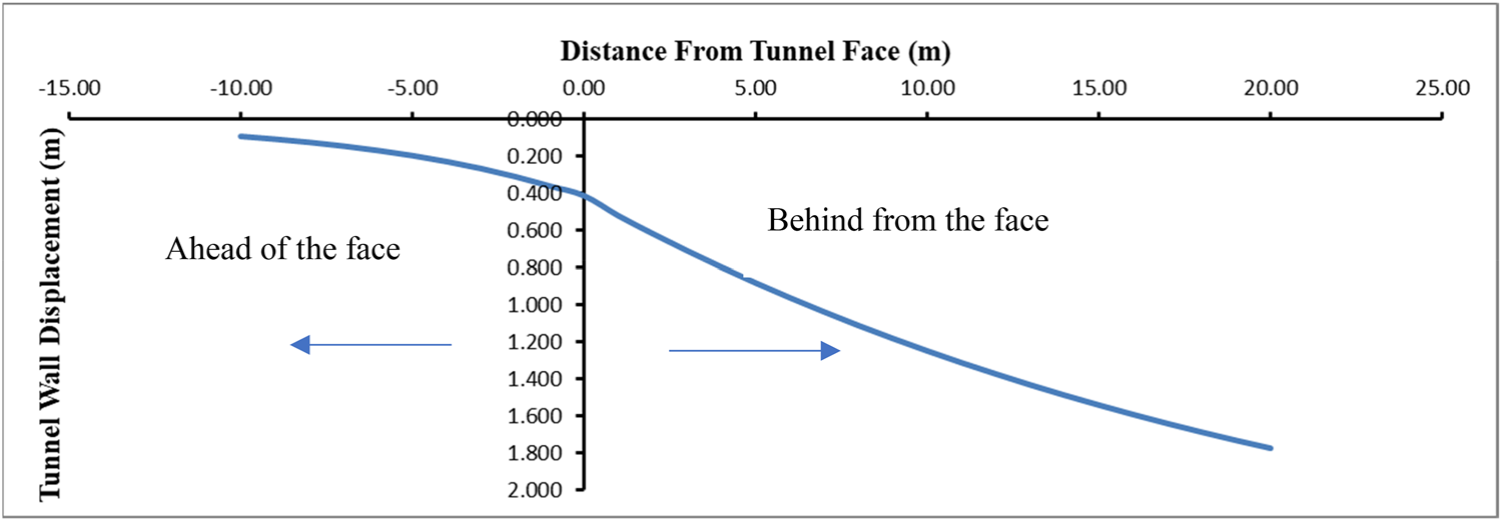
Longitudinal displacement profil for h = 200 m.

As can be seen, it is inevitable that tunnel stability cannot be achieved in the unsupported condition and serious face stability problems and deformations occurs. Many researchers have conducted studies to determine the deformations that occur in the tunnel^39–43^. Vlachopoulos and Diederichs^43^ equations (Eq. (14)) is used to determine the longitudinal dispaclacement profile along the tunnel. The longitudinal displacement profile for h = 200 m is given in Fig. 11.

14 $$u_{i} = \left\{ {\begin{array}{*{20}l} {u_{im} \cdot \left[ {\frac{{u_{if} }}{{u_{im} }} \cdot e^{{x/r_{0} }} } \right],} \hfill & {x < 0} \hfill \\ {u_{im} \cdot \left[ {\left( {\frac{{u_{im} }}{3}} \right)e^{{ - 0.15\left( {r_{pm} /r_{0} } \right)}} } \right],} \hfill & {x = 0} \hfill \\ {u_{im} \cdot \left[ {1 - \left( {1 - \frac{{u_{if} }}{{u_{im} }}} \right) \cdot e^{{\left( { - 3x/r_{0} } \right)/\left( {2r_{pm} /r_{0} } \right)}} } \right],} \hfill & {x > 0} \hfill \\ \end{array} } \right..$$

In the unsupported condition, 41 cm deformation occurs in the tunnel face, while this value increases to 52 cm, 1 m behind the tunnel. 20 m behind the tunnel, the deformations go up to 1.77 m.

As can be seen, serious deformations occur both in the tunnel face and in the tunnel behind the tunnel under 200 m of overburden height.

It is extremely important to determine the ground reaction curve and support reaction curves for the design of the support system in tunnels. Here, it is extremely important for the stability of the tunnel to apply the support at the right time^41,44–47^.

**Figure 12:**
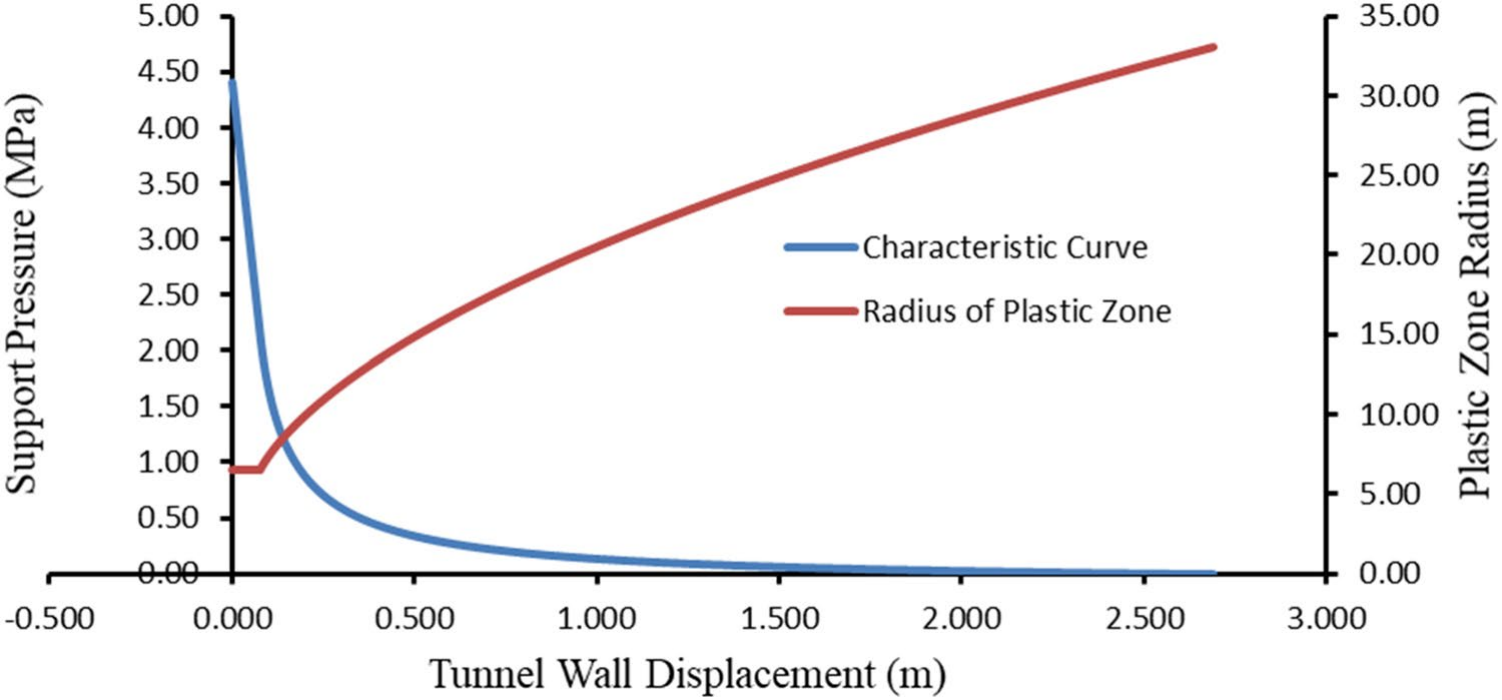
Longitudinal displacement profil for h = 200 m.

So, the variation of the soil reaction curve (GRC) and the radius of the plastic zone is given in Fig. 12. Here, it is seen that the radius of the plastic zone and the soil reaction curve develop very rapidly.

**Figure 13:**
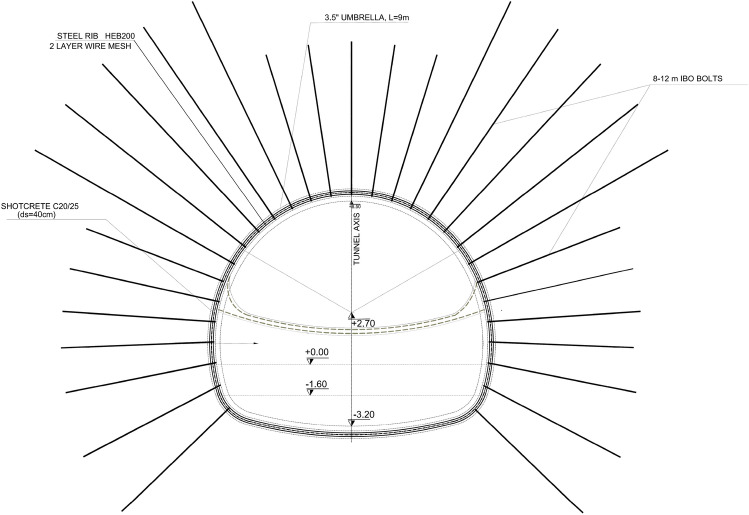
C3 support details.

The C3 support system had to be revised after the severe deformations failures in the support systems in the tunnel. Details of this support system are given in Fig. 13 and in Table 5. In this section, the analyses will be made according to the given support system. As a support system, 40 cm C20/25 shotcrete, HEB 200 steel rib (75–100 cm spacing) 8–12 m long self-drilling drives, 9 m long 3.5″ diameter umbrella, temporary inverted, double layer wire mesh (Q589/443). In addition, to ensure tunnel face stability, 10 cm shotcrete and Q221/221 type mesh steel will be applied after each excavation in the tunnel face with 9 m long self-drilling bolts. The excavation carried out by keeping the distance between the tunnel top heading and bench excavations at a minimum distance (approximately 4.0 m).

**Table 5:** C3 support system details.

Raund length	Shotcrete	Steel rib	Bolt	Wire mesh
0,750 m	40 cm (C20/25)	HEB200	8–12 m	Q589/443

Support reaction curve and ground reaction curve were drawn for outer lining elements.

The analyses were carried out according to the principle of placing the support elements immediately by allowing minimum deformation in the tunnel face. In a sense, a rigid support is considered.

For tunnel support systems, the equations given according to Hoek and Brown^7^ and Hoek^8^ are used. Equations are given in Table 6.

**Table 6:** Support capacity equation^7,8^.

**Steel set**			
	σ_*ys*_ is the yield strength of the steel (MPa) *E*_*s*_ is the Young modulus of the steel (MPa) *A*_*s*_ is the cross-sectional area of the section (m^2^)	$Pssmax=\frac{\text{As}\times \sigma ys}{sl\times lro}$	(15)
	*s*_*l*_ is the set spacing along the tunnel axis (m) *r*_*o*_ is the radius of the tunnel (m) *P*_*ssmax*_ is the maximum support pressure *K*_*ss*_ is the stiffness	$Kssmax=\frac{\text{Es}\times As}{sl\times lr{o}^{2}}$	(16)
**Rock bolts**			
	*d*_*b*_ is the rockbolt or cable diameter (m) *l* is the free length of the bolt or cable (m) *E*_*s*_ is the Young modulus of the bolt or cable (MPa)	$Psbmax=\frac{Tbf}{sl\times sc}$	(17)
	*s*_*c*_ is the circumferential bolt spacing (m) *s*_*l*_ is the longitudinal bolt spacing (m) *T*_*bf*_ is the ultimate bolt or cable load *P*_*sbmax*_ is the maximum support pressure *K*_*ss*_ is the stiffness	$Ksb=Es\times \pi \times \frac{d{b}^{2}}{4lslsc}$	(18)
**Concrete or shotcrete**			
	*σ*_*cc*_ is the uniaxial compressive of the concrete or shotcrete (MPa) *E*_*c*_ is the Young modulus of the concrete or shotcrete (MPa) *υ* is the Poisson ratio of the concrete or shotcrete	$Pscmax=\frac{\sigma cc}{s}\times \left[1-\frac{{\left(ro-tc\right)}^{2}}{r{o}^{2}}\right]$	(19)
	*t*_*c*_ is the thickness of the lining (m) *r*_*o*_ is the radius of the tunnel (m) *P*_*scmax*_ is the maximum support pressure *K*_*ss*_ is the stiffness	$Ksc=\left(Ec\times \frac{r{o}^{2}-{\left(r0-tc\right)}^{2}}{2\times \left(1-{\vartheta }^{2}\right)\times \left(r0-tc\right)\times r{o}^{2}}\right)$	(21)

The support pressure, support stiffness and maximum displacement are given in Table 7.

**Table 7:** Outer lining and inner lining support system pressures.

Support types	*p*_*scmax*_ (MPa)	*K*_*sc*_ (MPa/m)
Shotcrete (ds = 40 cm)	1.192	285.2
Steel rib (HEB200)	0.585	51.00
Rock bolts	0.373	27.74

The ground and support reaction curves are drawn with the RocSupport program. Maximum support pressure and stiffness values are given below.

$${\text{Max}}.{\text{outer lining support pressure}}:{\text{ P}}_{{{\text{outmax}}}} = {\text{ P}}_{{{\text{ssmax}}}} + {\text{P}}_{{{\text{scmax}}}} + {\text{P}}_{{{\text{sbmax}}}} = {1}.{192} + 0.{585} + 0.{373} = {2}.{\text{15 MPa}},$$

$${\text{Max}}.{\text{ stiffness}}:{\text{ K}}_{{{\text{outer}}}} = {\text{ K}}_{{{\text{sb}}}} + {\text{K}}_{{{\text{st}}}} + {\text{K}}_{{{\text{sc}}}} = {285}.{2} + {51}.0 + {27}.{74} = {363}.{\text{94 MPa}}/{\text{m}}.$$

**Figure 14:**
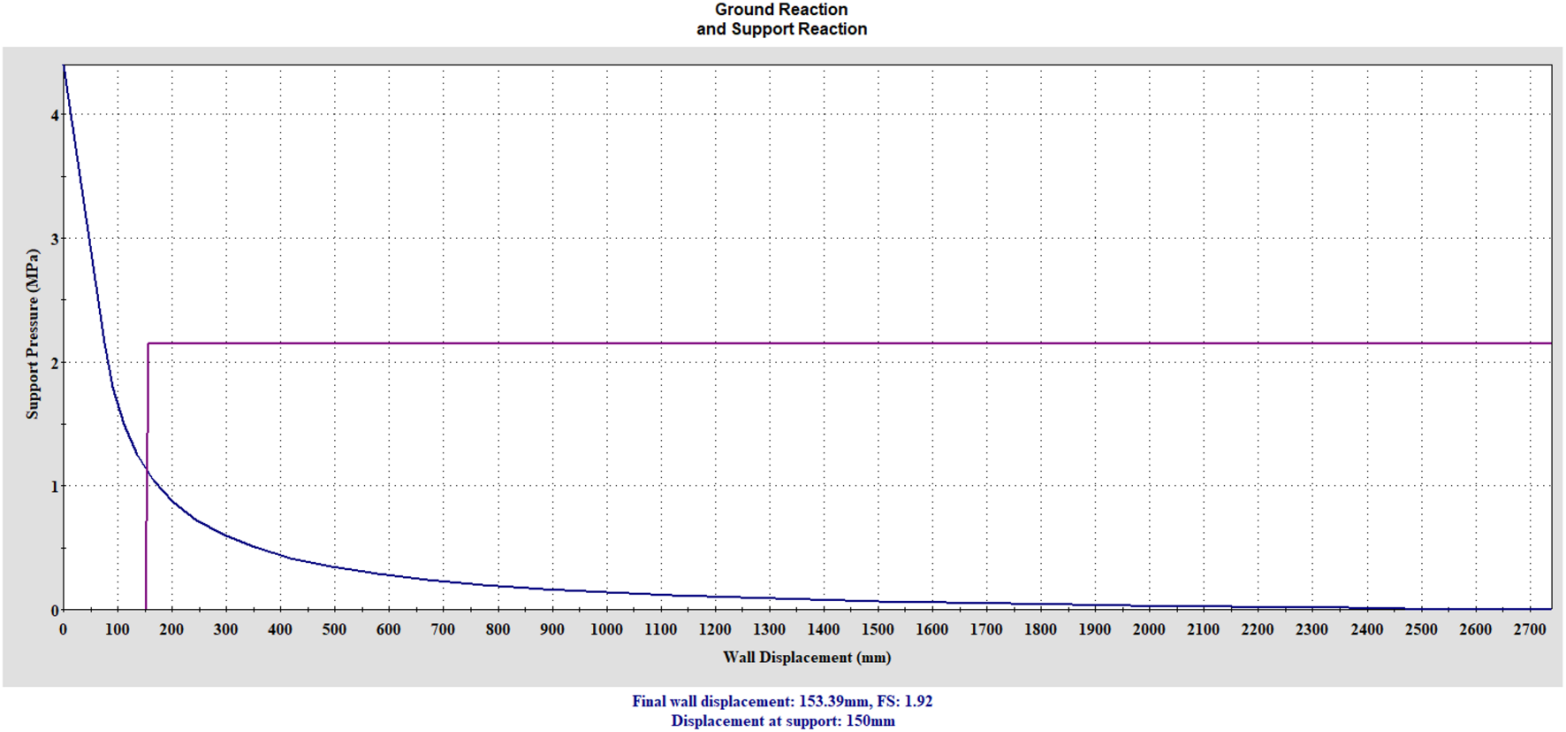
Outer lining ground and support reaction curves.

The summary table of the analyses made for this section of the tunnel is presented in Table 8. Figure 14 presents the ground and support system reaction curves against the total external support pressure for the outer lining. As can be seen here, the safety factor is calculated as 1.92 and the convergence occurring in the tunnel is 2.36%.

**Table 8:** Summary of the analysis result.

	Outer lining
Support pressure (MPa)	2.15
Factor of safety	1.92
Mobilized support pressure (MPa)	1.12
Tunnel convergence (%)	2.36
Critical pressure (MPa)	2.16

According to the analyses results, tunnel stability is ensured by the applied support systems. However, it is seen that the main reason for the collapse and occurred deformations is the excavation and support sequence could not be achieved in the real time. It is seen that deformations occurred due to the fact that the ring was not closed in the top heading excavations, which were kept for a long time. The resulting deformations increase rapidly and cause collapse.

## 3D numerical analyses

Flac3d^48^ program is used to model the deformation and collapse in the tunnel. These analyzes are carried out to see why the collapse occurred in the 28 m section between km: 62 + 910 and km: 62 + 938. Except for the section where the collapse occurred, the collapse did not occur even though the deformations were outside the tolerances. However, after the top heading was completed, a collapse occurred in the 28 m section where the bench and invert excavations were not completed. This shows us that if the ring is closed, there will be no problem.

**Figure 15:**
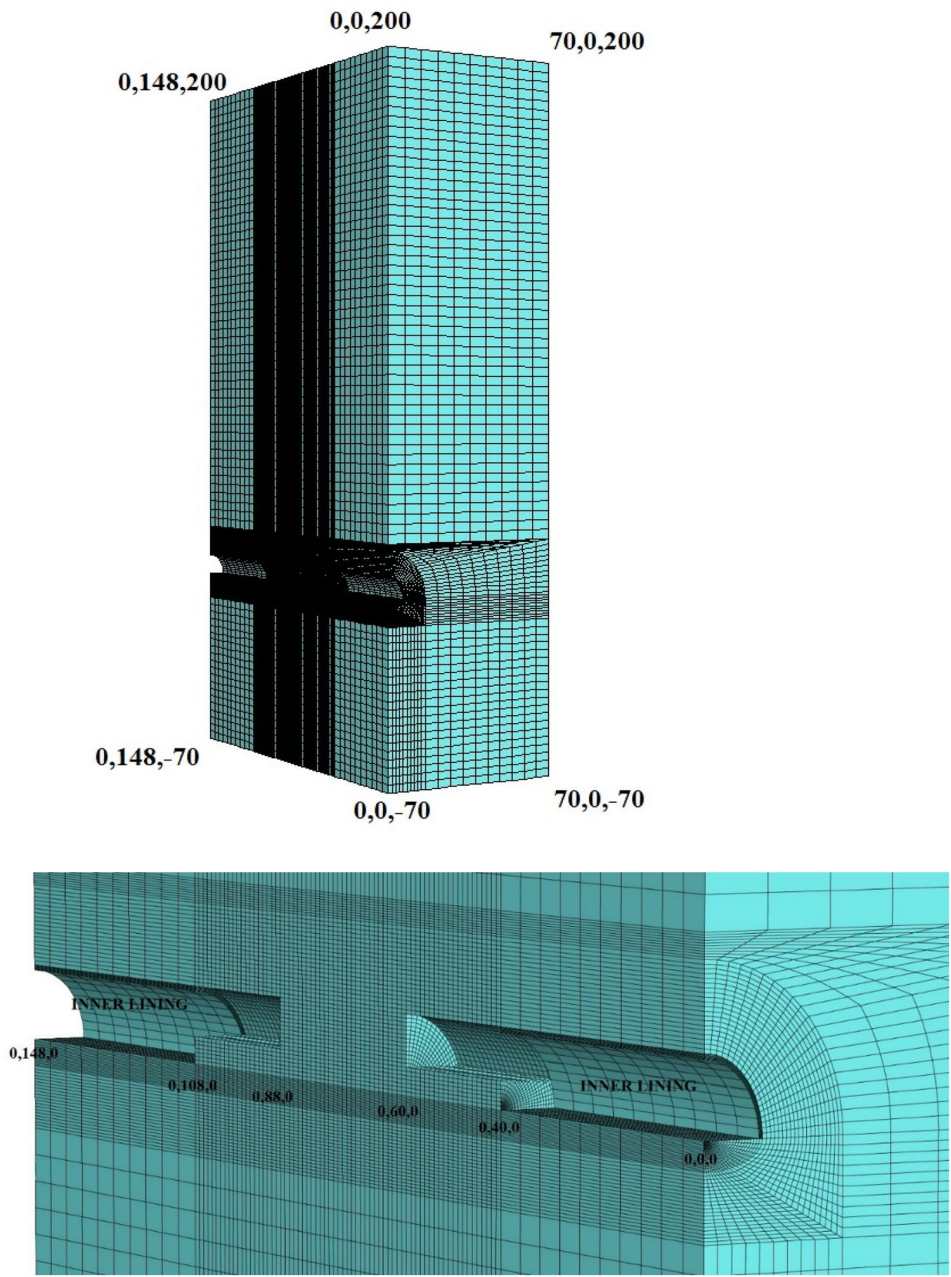
Flac3d model.

**Figure 16:**
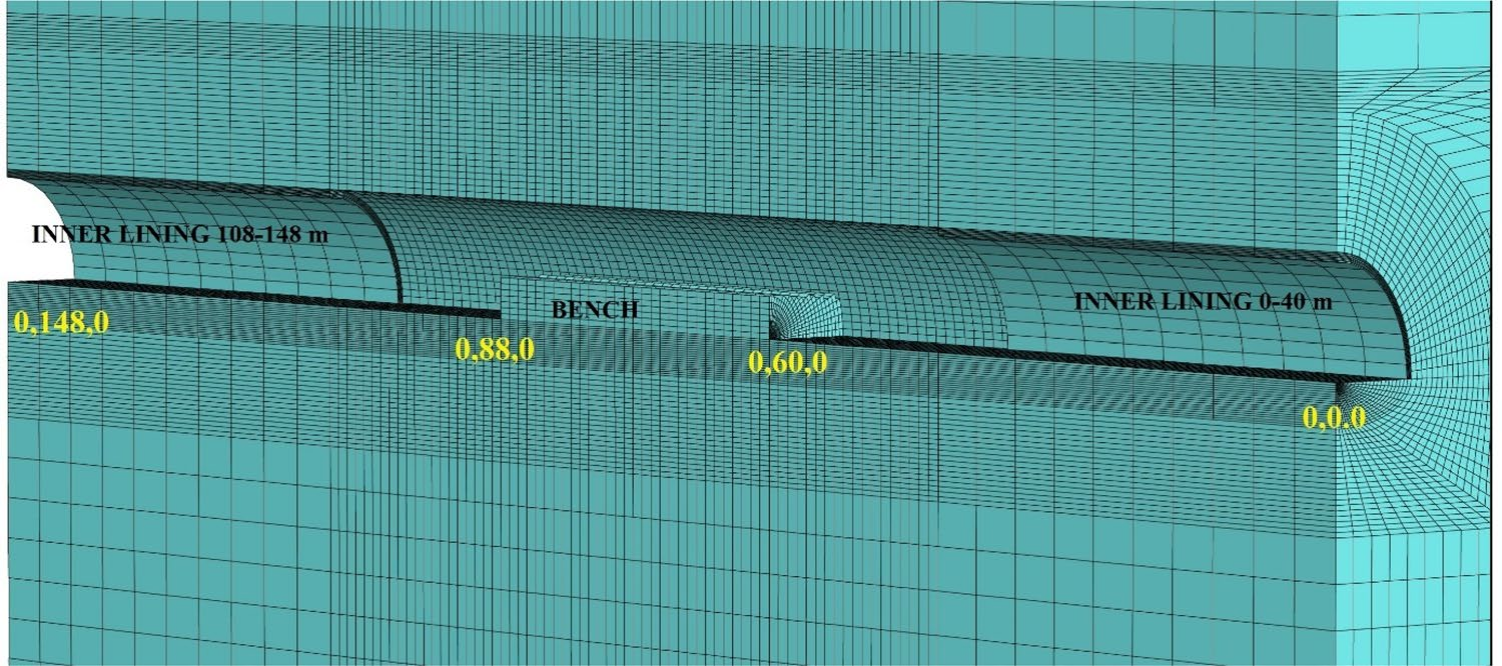
Excavation steps.

During the modeling stages, the model is prepared by taking 148 m in the Y direction, 70 m in the X direction, and 200 m in the Z direction (Fig. 15). While the excavation stages are created in the model, excavation is carried out from both entrances. As in the field applications, the top heading, bench and invert excavations of the first 40 m of the entrance and exit sections (between 0–40 and 148–140 m) have been completed and the model has been entered by making the inner lining concrete (Fig. 16). The model is solved at this stage, and then the deformations are reset. In the next stages, the tunnel is excavated in the form of the top heading, the bench, and the invert, and the model is continued to be solved by making it from both entrances. Between km: 62 + 910 and km: 62 + 938, which is the area where the collapse occurred, only the top heading is excavated, and the bench and inverted excavations are not completed. In this case, the deformations in the tunnel are investigated. The model is solved in a total of 73 steps.

**Figure 17:**
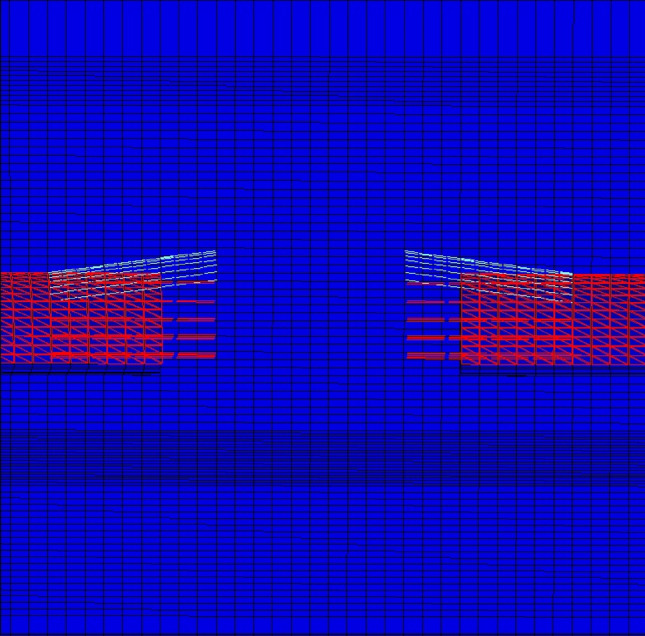
Face bolts and umbrella systems in the top heading section.

The parameters used in the model are given in Table 1. Flac3d program uses bulk modulus and shear modulus in solutions. The Bulk Modulus (K) value was calculated as 208 MPa and the shear modulus (G) value was calculated as 96 MPa. Mohr–Coulomb failure criterion was chosen in the analysis and the model was solved by the gravity method. The model is assumed to be symmetrical and only half of the tunnel is modeled. In the analyses, 40 cm C20/25 type shotcrete was defined in the model as a shell element, and 88.9 mm diameter and 9 m long umbrellas on the tunnel ceiling were defined as pile elements in the model. In addition, 9 m long bolts in the tunnel mirror were entered into the model as cable bolts. Both umbrella and cable bolts were applied during excavation phases in the model with a 4.5 m thrust (Fig. 17).

The material parameters for shotcrete, cable bolt, and umbrella used in the model are presented in Tables 9, 10, and 11. 248,400 zone and 262,567 gridpoints, 3956 structural elements, and 2536 nodes are used in the model.

**Table 9:** Parameters used for shotcrete lining concrete.

Element	Ei (GPa)	√	ϒ (kg/m^3^)	Ds (cm)
Shotcrete	30	0.25	2500	40

**Table 10:** Parameters used for cable bolts.

Cable modulus (GPa)	Cable area (m^2^)	Cable ultimate tensile capacity (kN)	Grout bond stiffness (N/m/m)	Grout cohesive strength (N/m)
45	0.85 × 10^−3^	250	1.75 × 10^7^	2.0 × 10^5^

**Table 11:** Parameters used for umbrellas.

Diameter of pipe (m)	Wall thickness of pipe (m)	υ	E (GPa)	Yield (N)	Cross sectional area (m^2^)	Perimeter
0.114	0.0065	0.3	200	200,000	0.006204	0.279

Modeling stages are given in Table 12.

**Table 12:** Modelling stages.

Stage	Excavation situation	Stage	Excavation situation
1	Unbalanced forces	52–53	Excavation between 92–90 and 90–88 m in bench
2	Excavation between 0–40 and 140–148 m full face excavation and 40–60 m and 140–100 m top heading excavation, installation supports and installation inner lining concrete	54	Excavation between 58 and 60 m in bench
3–4	Excavation between 60–61 and 61–62 m in TH	55	Excavation between 88 and 86 m in bench
5–6	Excavation between 40–42 and 42–44 m in bench	56	Excavation between 44 and 48 m in invert
7	Excavation between 36 and 40 m in invert	57	Excavation between 100 and 96 m in invert
8–9	Excavation between 62–63 and 63–64 m in TH	58	Excavation between 48 and 52 m in invert
51	Excavation between 56 and 58 m in bench	59	Excavation between 95 and 92 m in invert
10	Excavation between 44 and 46 m in bench	60	Excavation between 52 and 56 m in invert
11–12	Excavation between 64–65 and 65–66 m in TH	61	Excavation between 92 and 88 m in invert
13	Excavation between 46 and 48 m in bench	62	Excavation between 62 and 60 m in bench
14	Excavation between 40 and 44 m in invert	63	Excavation between 86 and 84 m in bench
15–16	Excavation between 88–87 and 87–86 m in TH	64	Excavation between 62 and 64 m in bench
17–18	Excavation between 108–106 and 106–104 m in bench	65	Excavation between 84 and 82 m in bench
19	Excavation between 108 and 104 m in invert	66	Excavation between 64 and 66 m in bench
20–21	Excavation between 86–85 and 85–84 m in TH	67	Excavation between 82 and 80 m in bench
22	Excavation between 104 and 102 m in bench	68	Excavation between 68 and 70 m in bench
23–24	Excavation between 83–84 and 83–82 m in TH	69	Excavation between 80 and 78 m in bench
25	Excavation between 102 and 100 m in bench	70	Excavation between 70 and 72 m in bench
26	Excavation between 100 and 104 m in invert	71	Excavation between 78 and 76 m in bench
27–34	Excavation between 66–67 m, 67–68 m, 68–69 m, 69–70 m, 70–71 m, 71–72 m, 73–74 m in TH	72	Excavation between 72 and 74 m in bench
35–42	Excavation between 81–82 m, 81–80 m, 80–79 m, 79–78 m, 78–77 m,77–76 m, 76–75 m, and 75–74 m in TH	73	Excavation between 76 and 74 m in bench
43	Excavation between 48 and 50 m in bench		
44	Excavation between 100 and 98 m in bench		
45	Excavation between 50 and 52 m in bench		
46	Excavation between 98 and 96 m in bench		
47	Excavation between 52 and 54 m in bench		
48	Excavation between 96 and 94 m in bench		
49	Excavation between 54 and 56 m in bench		
50	Excavation between 94 and 92 m in bench		
51	Excavation between 56 and 58 m in bench		

### Evaluation of analysis results

During the evaluation of the analysis results, it has been evaluated in four parts to see the interaction of the top heading, bench, and invert. In the first part, the section where the excavations continue consecutively from both faces of the tunnel is examined. This section includes 26 levels as given in Table 12. Until this stage, successive excavations between 60 and 66 m in the top heading, between 40 and 48 m in the bench, and between 36 and 44 m in the invert section are carried out from the tunnel excavation entrance side. In the exit face section, between 88 and 82 m in the top heading, between 108 and 100 m in the bench, and between 88 and 80 m in the invert section, excavations and supports are carried out consecutively.

“Tunnel specifications” section, it covers stages 26 to 42. Here, only the top heading area is excavated, and the top heading excavation is completed. This section covers between 82 and 108 m.

In “Evaluation of tunnel squeezing conditions and tunnel behaviour” section, excavations between 80 and 88 and 48 and 60 m are excavated on the bench in accordance with the current situation in the excavation phase in the tunnel. At this stage, the 28 m section in the bench is not excavated. This situation represents the pre-collapse situation in the tunnel.

In “Analytical solutions and tunnel support reaction curves” section, the tunnel invert concrete is completed and then the bench excavations between 60 and 88 m are completed in both directions of progress and the status of the tunnel being completed is examined.

#### *Evaluation of the section 1 analysis*

**Figure 18:**
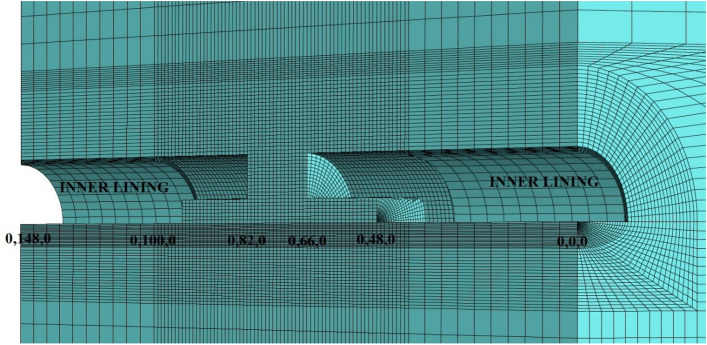
Tunnel excavation situation after stage 26.

**Figure 19:**
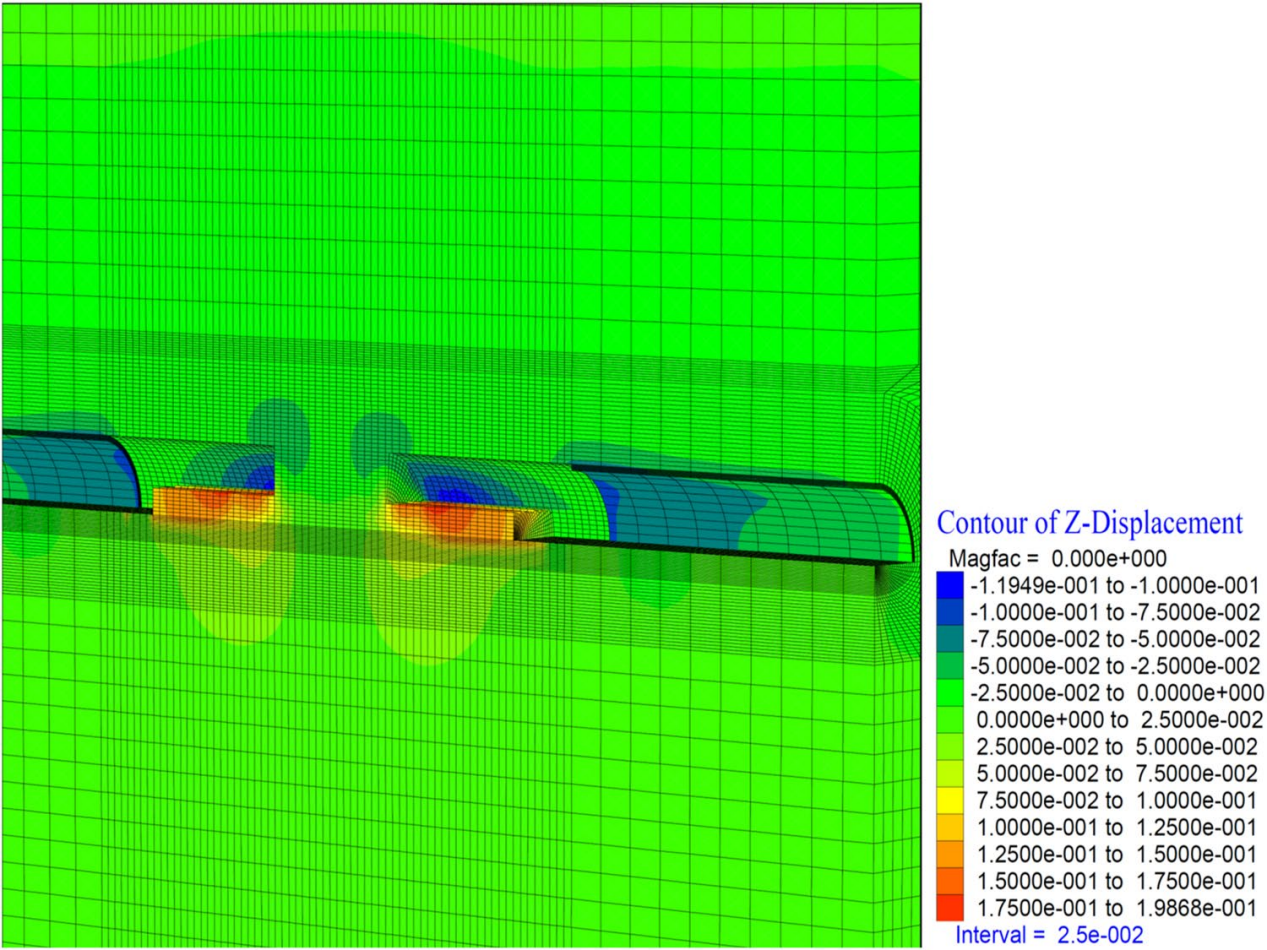
Z displacement in stage 26.

**Figure 20:**
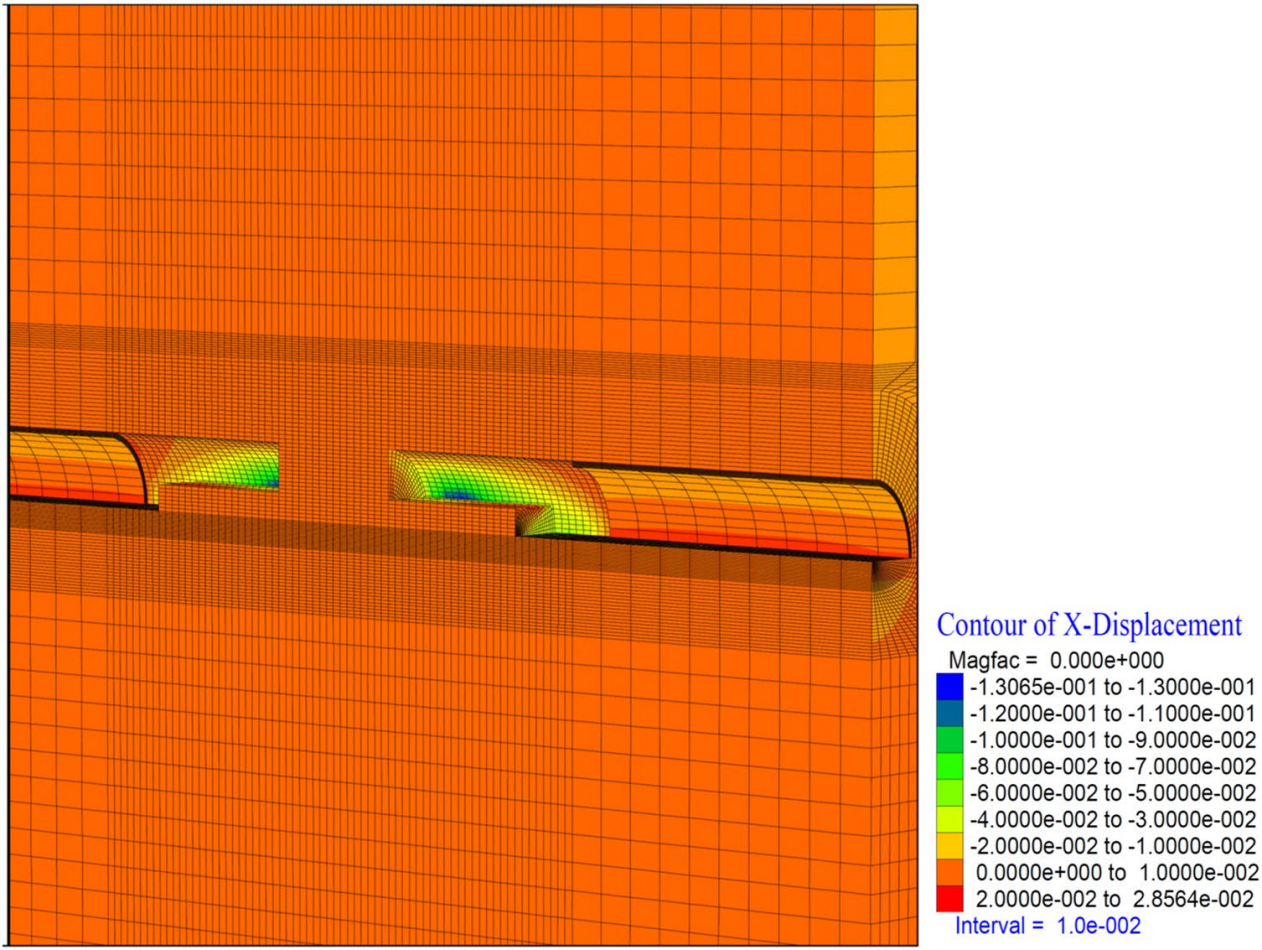
X displacement in stage 26.

**Figure 21:**
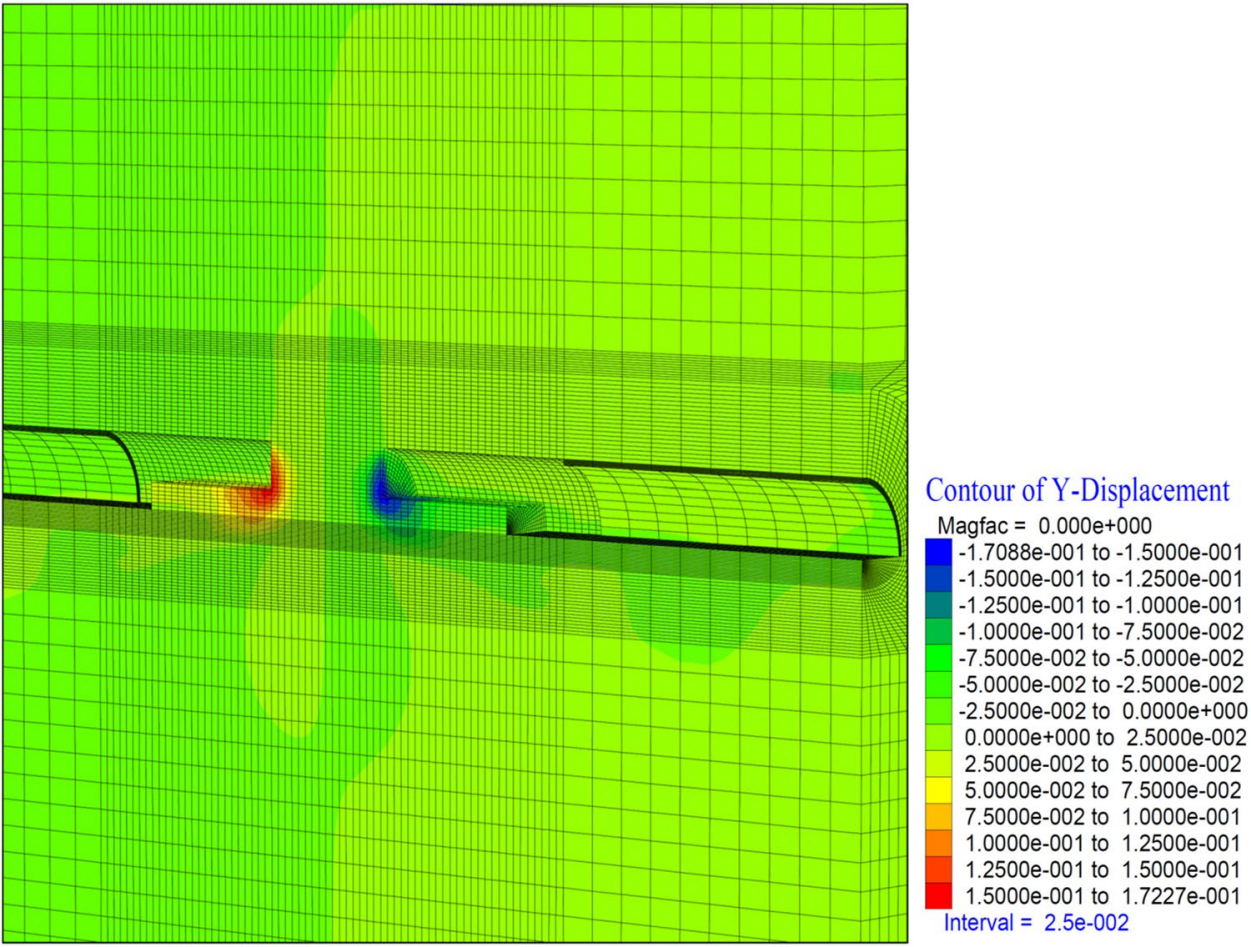
Y displacement in stage 26.

In these stages, the tunnel is excavated in the top heading, bench, and inverted form, and the ring is immediately closed (Fig. 18). Deformation values in consecutive excavations are given in Figs. 19, 20 and 21.

In this case, vertical deformations in the tunnel (in the Z direction) occurred at the level of 11 cm. In the top heading, a displacement of 17.5 cm occurred in the form of a squeezing at the base part (Fig. 19). In the region where the inner lining of the tunnel is completed, deformations are observed at the level of 11 cm. This section, where the inner lining is completed, is 26 m away from the tunnel face. In a sense, deformations in the 2D distance (tunnel diameter 13 m) behind the tunnel face showed their effect. Theoretically, the impact distance of the tunnel excavation is suitable for 2D. If the inner lining concrete is to be constructed at a 2D distance from the tunnel face, it should be constructed in such a way as to bear these deformations and stresses that occur due to the tunnel excavation.

Similarly, closure is observed at the level of 13 cm in the horizontal (in the X direction) (Fig. 20). It is seen that these deformations are concentrated 10 m behind the tunnel face. Horizontal deformations are reduced and stabilized behind the 2D distance of the tunnel. In a sense, closing the ring 2D behind the tunnel diameter resets the horizontal deformations.

The deformations in the tunnel face towards the tunnel excavation direction occurred at a maximum level of 17 cm (Fig. 21). These values occurred in the edge sections of the model and stabilized in the middle measurement of the tunnel face. It is observed that the forepolings and face bolts are sufficient.

#### *Evaluation section 2 analysis*

**Figure 22:**
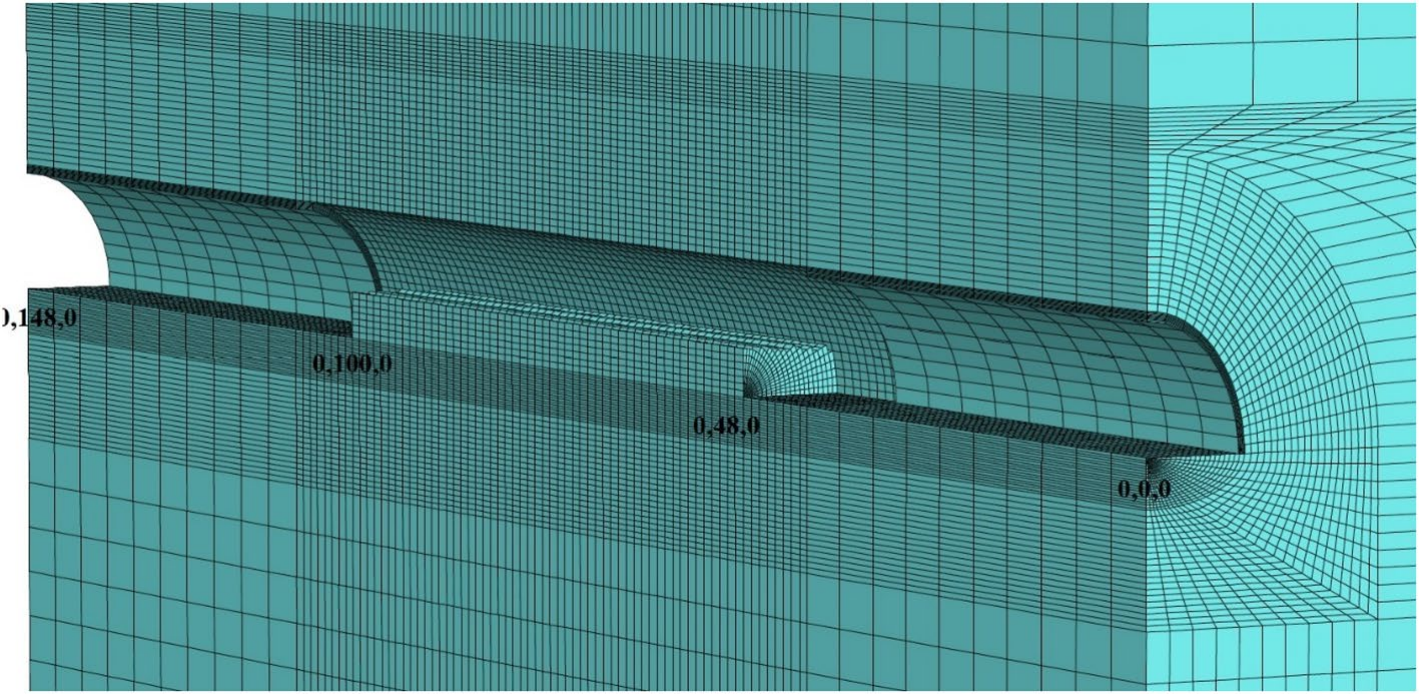
Tunnel excavation situation after stage 42.

In this part of the analysis, only the top heading of the tunnel is excavated, and the top heading of the tunnel is opened (Fig. 22). In this section, it is aimed to examine the stability of the tunnel without closing the ring, that is, without the bench and invert excavations. As seen in Table 12 during the modeling stages, these excavations are completed at stage 42.

**Figure 23:**
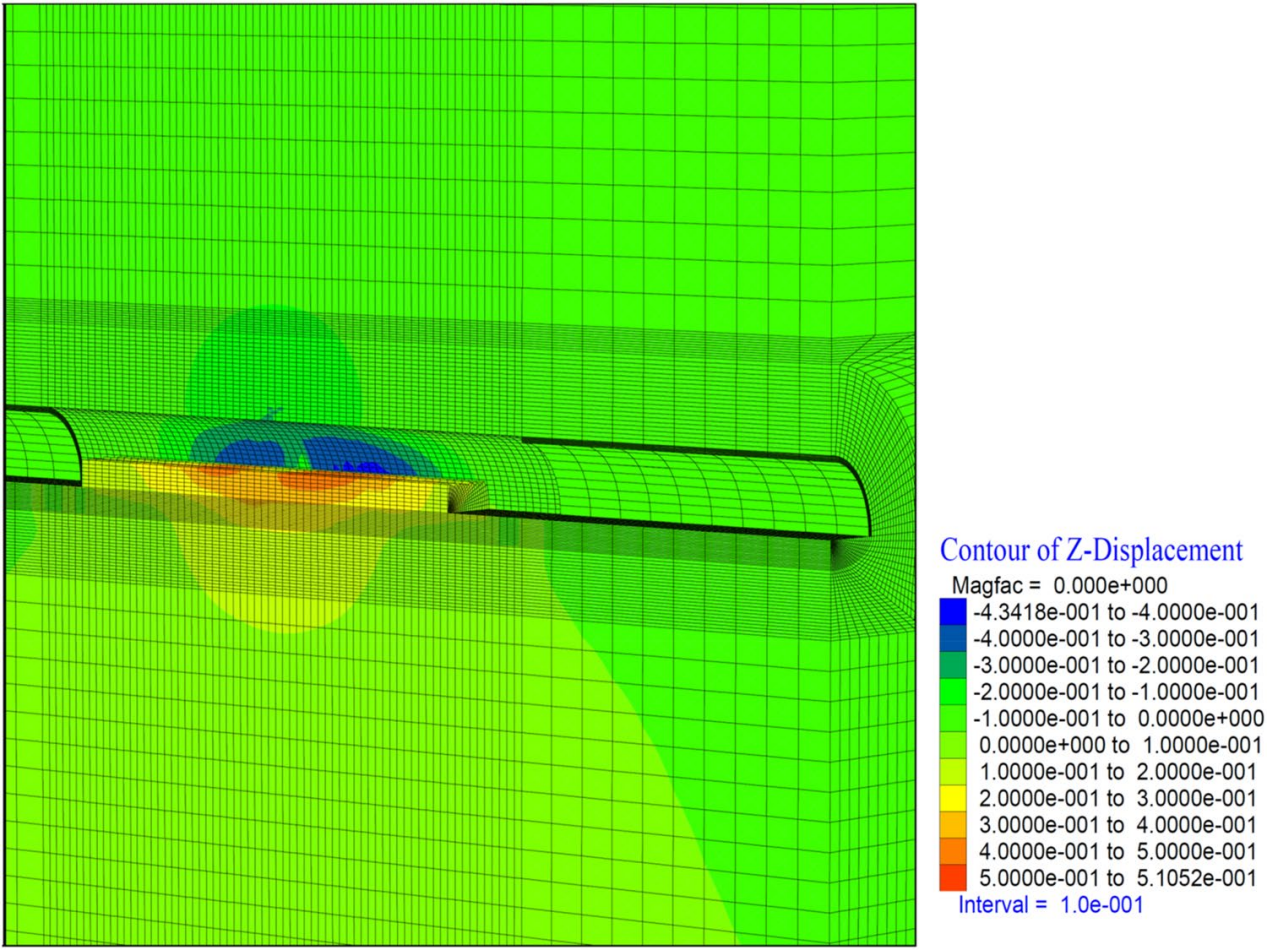
Z displacement in stage 42.

When the deformations occurring in the top heading of the tunnel are examined, it is seen that the deformations in the vertical direction reach up to 43 cm. In the top hearing, it is determined that the deformations reached 50 cm in the form of swelling (Fig. 23). In a sense, it is seen that the tunnel has completely lost its stability and the supports have been yielded. Behind the tunnel, it is seen that the deformations do not increase, as the tunnel excavation has no effect on the inner lining concrete. In other words, it is understood that the deformations lose their effect in the section where the tunnel face excavation exceeds 2D.

**Figure 24:**
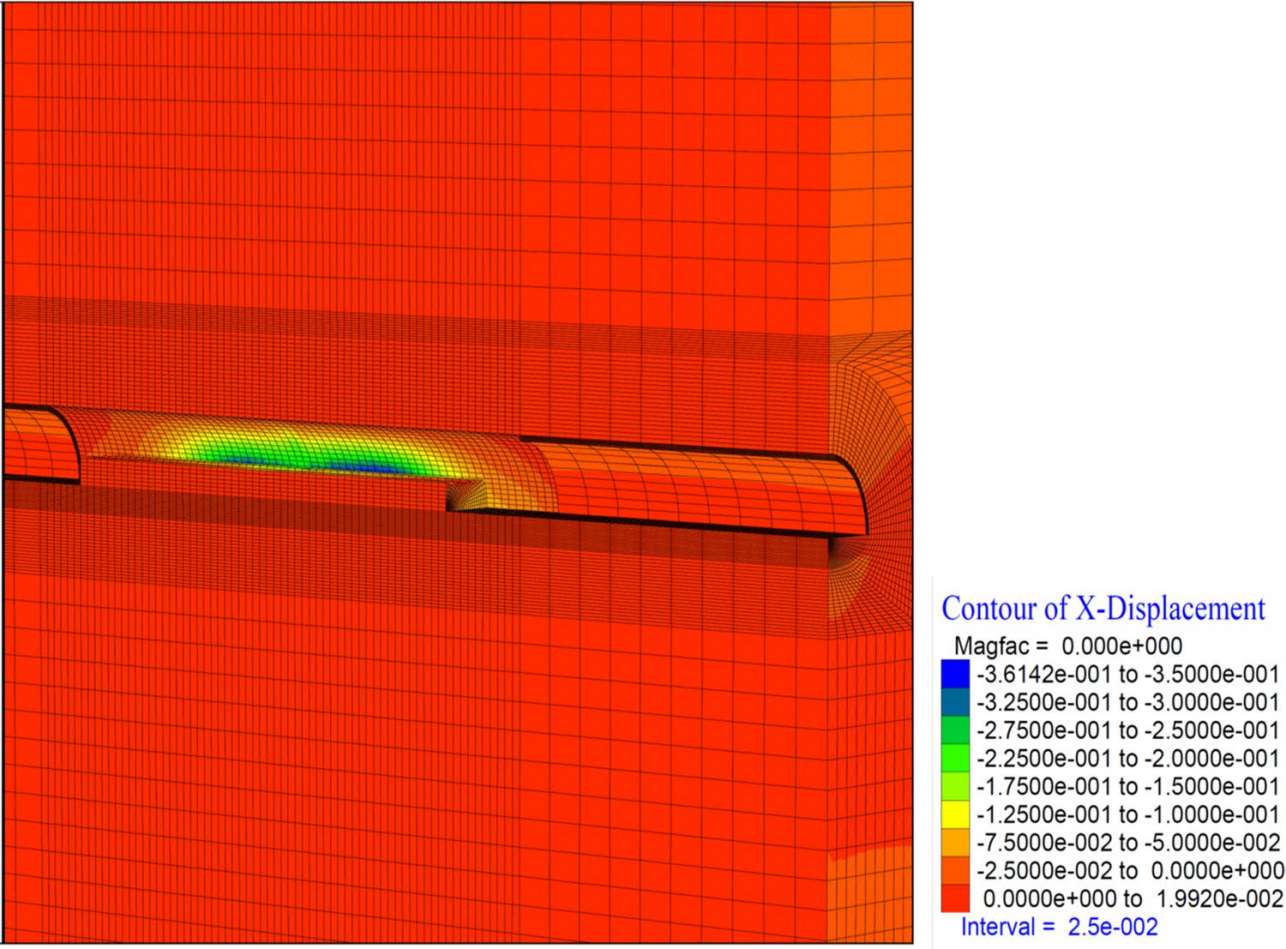
X displacement in stage 42.

It is determined that the deformations in the tunnel reached up to 36 cm in the horizontal direction (in the X direction) (Fig. 24). It is observed that these deformations are concentrated at the intersection of the top heading and the bench and continued throughout the top heading.

#### Evaluation of section 3

**Figure 25:**
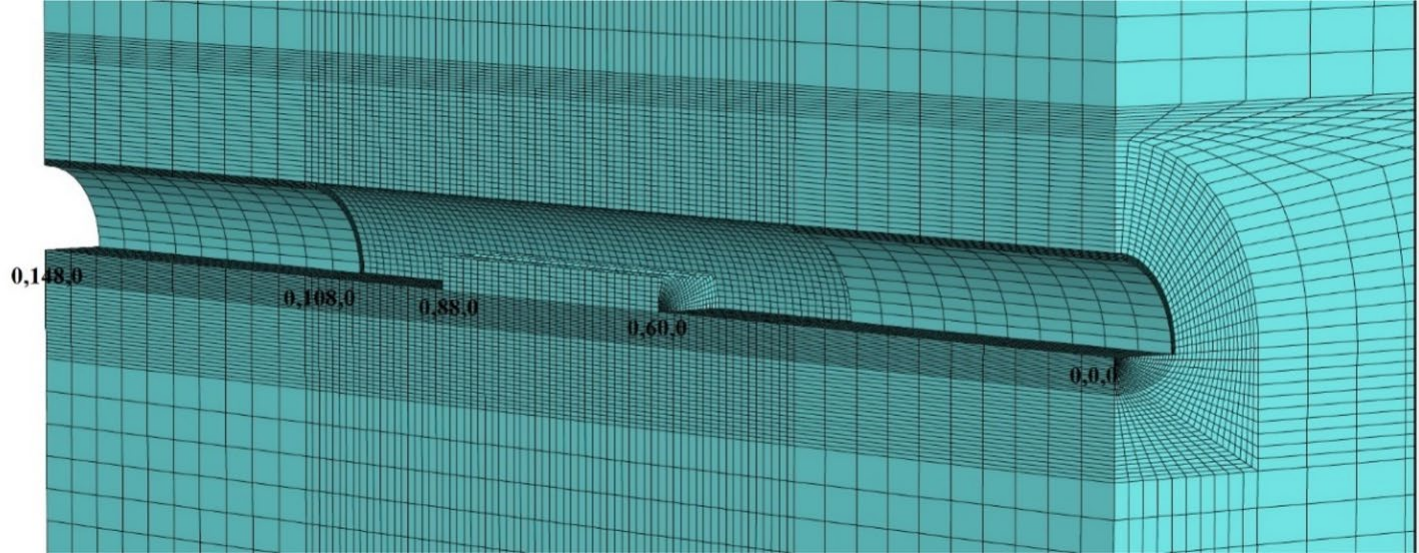
Tunnel excavation situation after stage 53.

In the third stage, the situation in which the excavation of the bench excavation is examined. This situation represents the situation at the collapse point of the tunnel. In this section, the tunnel is formed in a 28 m section as the unexcavated distance in the top heading (Fig. 25). For this case, excavations are carried out between 80 and 88 m and 48 and 60 m in the benches.

**Figure 26:**
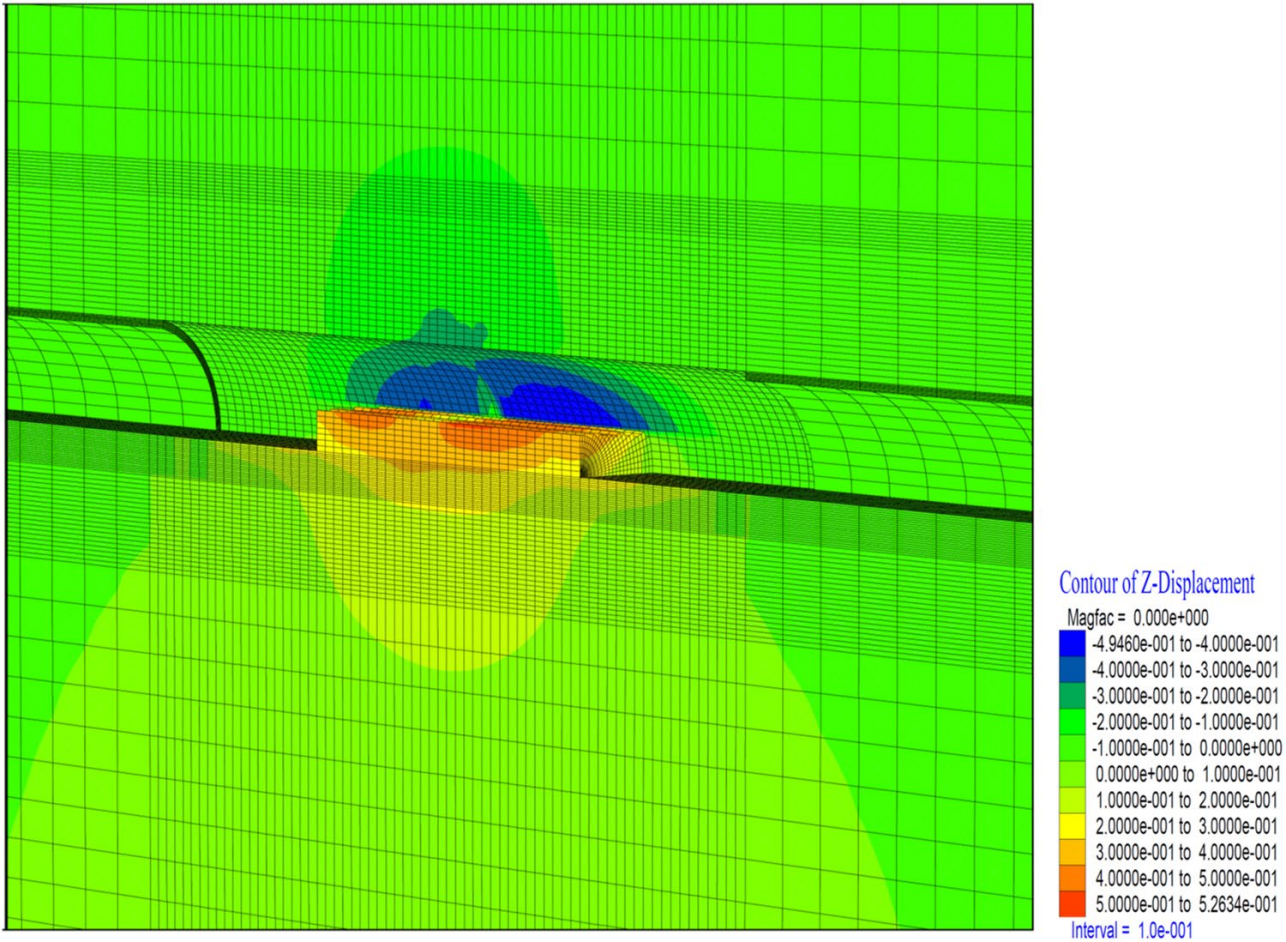
Z displacement in stage 53.

**Figure 27:**
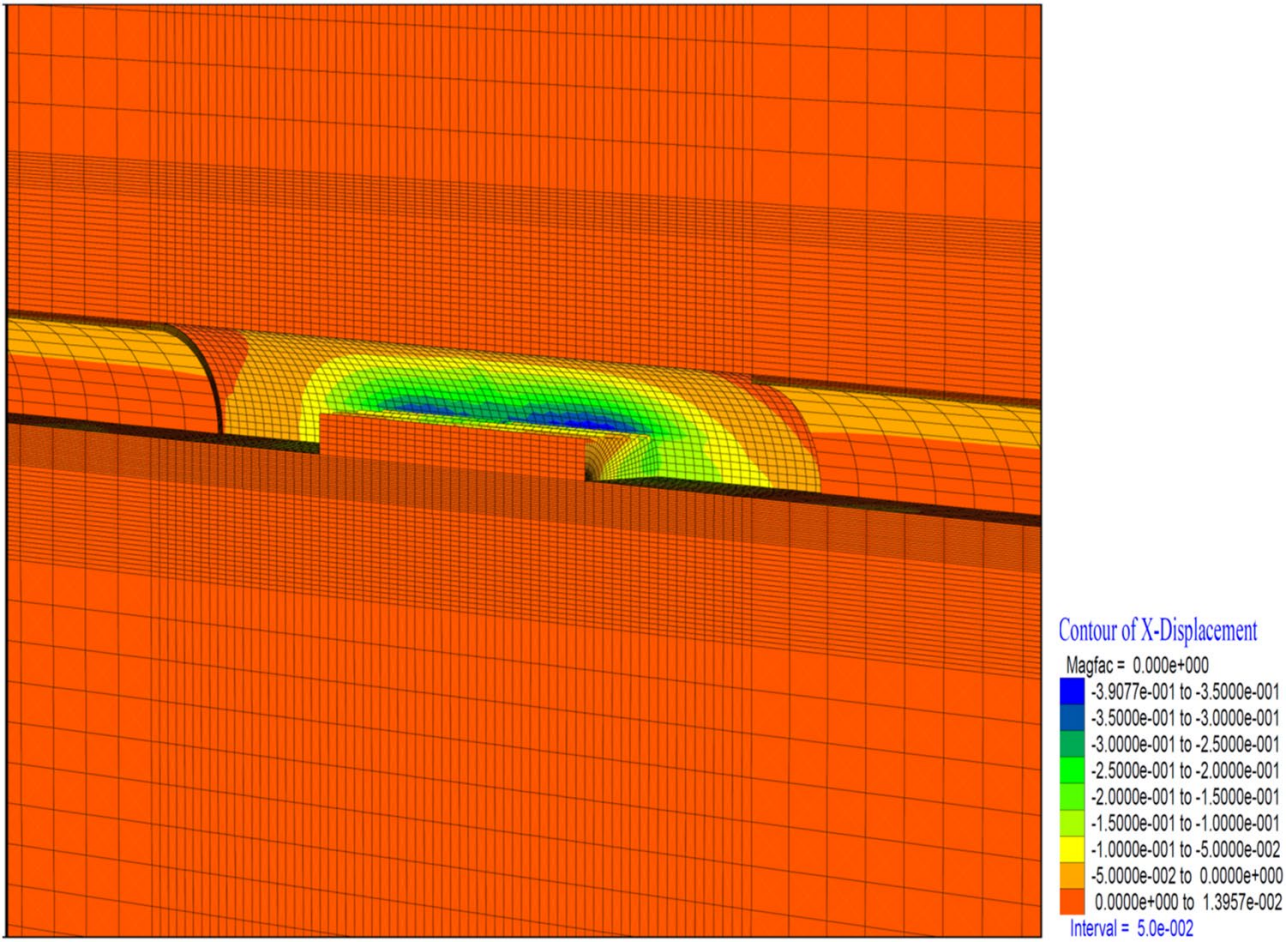
X displacement in stage 53.

As the excavation stage, the excavation process is terminated in 53 stages (Table 12). It is observed that the deformations occurring in the vertical direction in the tunnel increased with the excavations in the bench, and deformations up to 50 cm occurred in both the ceiling section and the top heading section. In a sense, up to 1.0 m of closure has occurred (Fig. 26). On the side walls of the tunnel, the deformations in the X direction reached 40 cm. In other words, after the excavations in the top heading, along with the excavations in the bench, the deformations in the X direction increased by 4 cm in total (Fig. 27).

#### *Evaluation of section 4*

**Figure 28:**
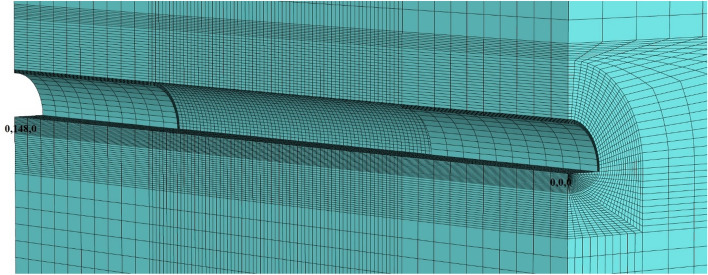
Tunnel excavation situation after stage 74.

In this part of the analysis, the situation in which the bench of the tunnel is excavated examined and analyzes are performed with the final finished state of the tunnel (Fig. 28).

**Figure 29:**
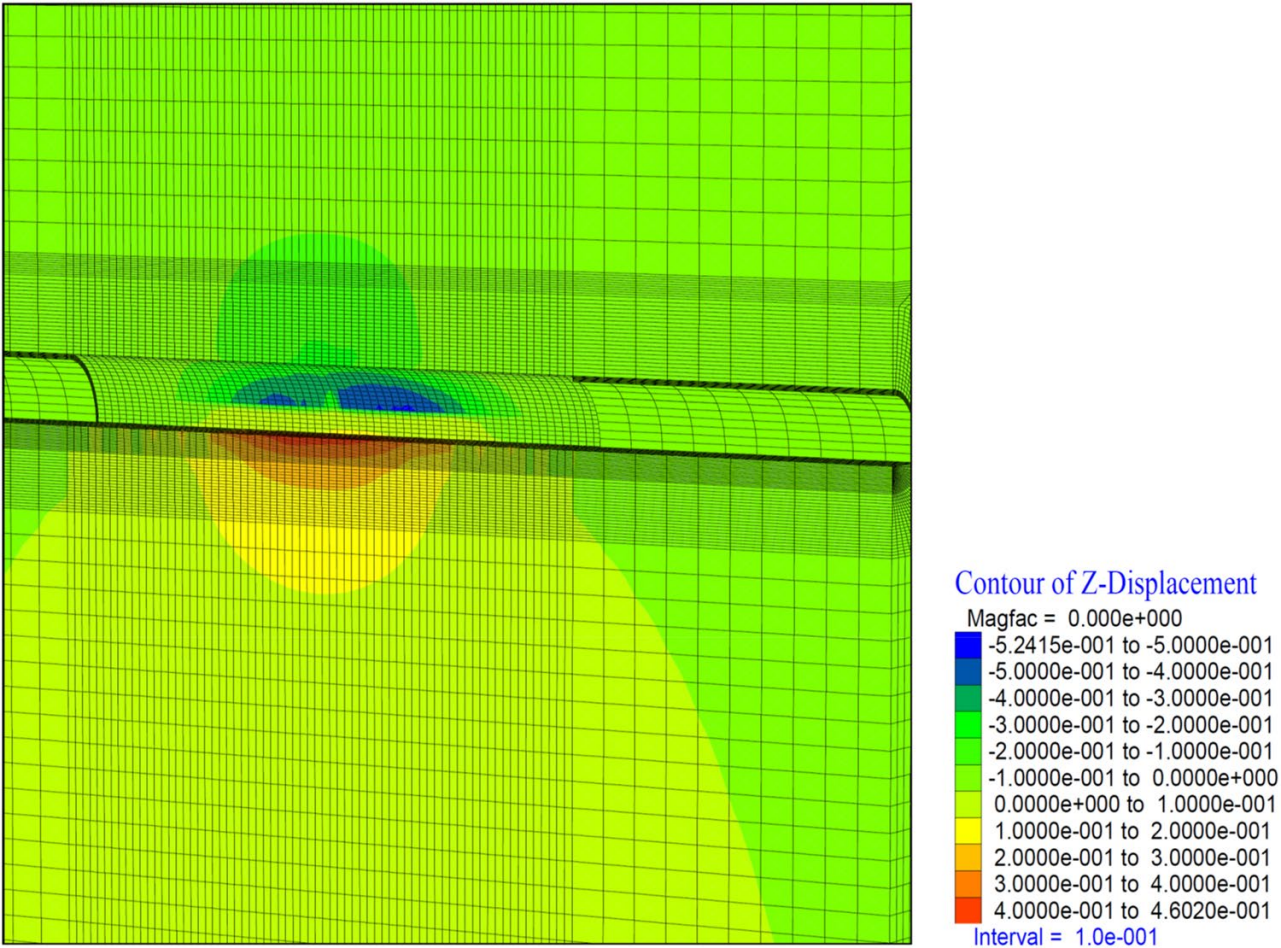
Z displacement in stage 74.

**Figure 30:**
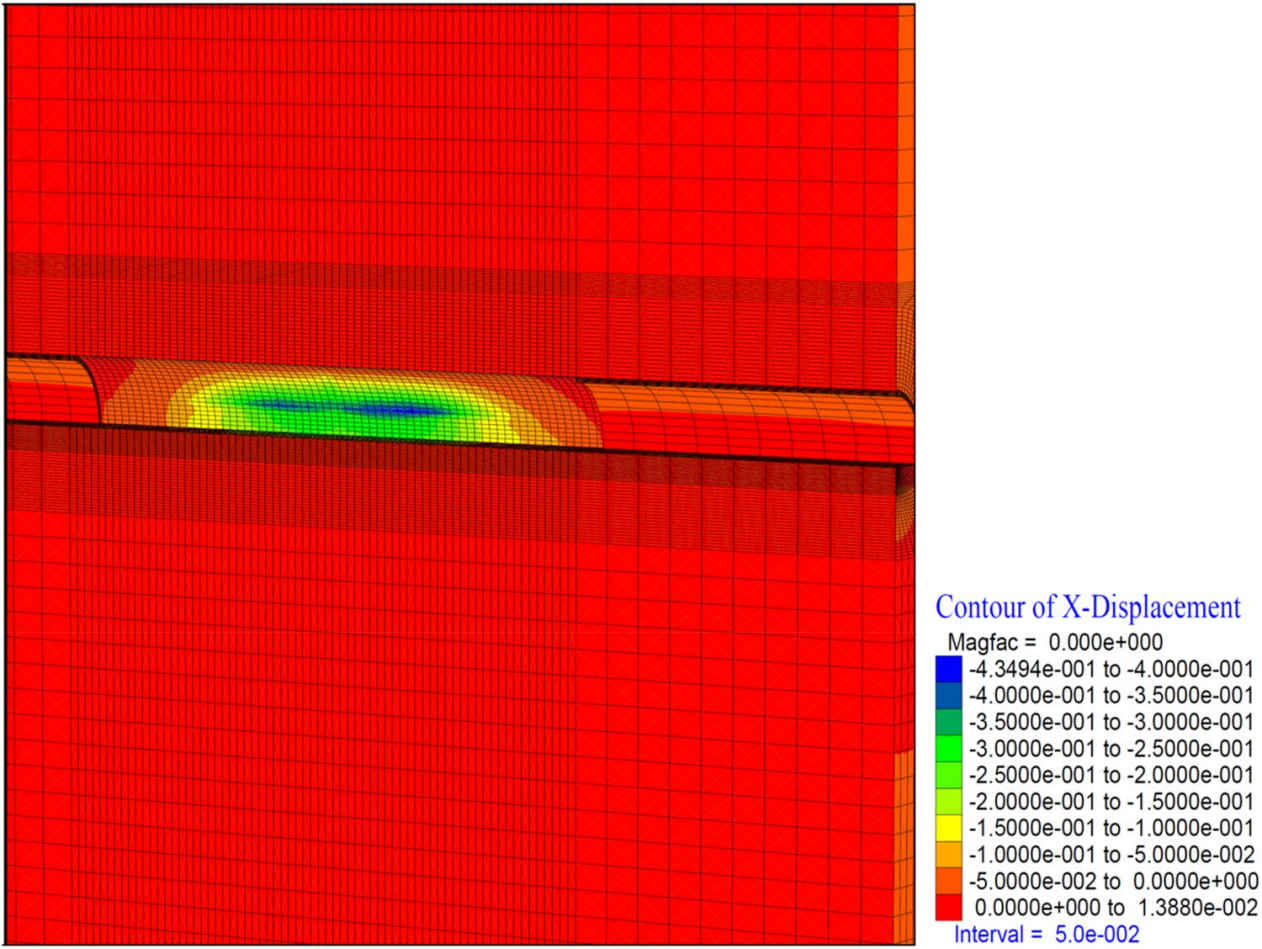
X displacement in stage 74.

In theanalysis, it is seen that the deformations in the vertical direction (Z direction) in the tunnel increase up to 52.4 cm. In the base part, squeezing deformations up to 46 cm were formed (Fig. 29). With the deformations occurring in the horizontal direction (X direction) in the tunnel, closure up to 44 cm occurs (Fig. 30) In this case, after the completion of the excavation in the top heading of the tunnel (stage 53), there is a displacement of 4 cm with the excavations on the bench. As can be seen here, the main deformations occur only in the upper half of the tunnel without the lower half excavation, that is, without closing the ring. While the deformations that occurred at stage 26, where gradual excavation is carried out, were 13 cm, it increased to 40 cm in the case of excavation only in the top heading (stage 42). Deformations increase 3 times.

## Conclusion

3D numerical analyzes are performed for the T6 tunnel. In the analysis, the existing collapsed section is modeled according to the support systems applied in the field.

In the analysis, it has been seen that it is appropriate to perform the sequential excavation in the form of the tunnel top heading, bench, and invert.

It is determined that the deformations increased 3 times in cases where the excavation is only performed in the top heading. In this case, it is determined that the support systems in the tunnel were yielded and the deformations increased to 40 cm in the top heading side walls and invert section. It is revealed in the analysis that the total closure that occurred reached 80 cm.

It has been determined that the squeezing mechanism causes serious problems in tunnels excavated on weak grounds, especially in schists. In these cases, the ring should be closed after 30 m behind the face. The inner lining concrete should be considered as a load-bearing element. Otherwise, the outer lining starts to yield in the long term. It has been determined that keeping a minimum distance between the top heading, bench and invert excavations is extremely important for the stability of the tunnel.

Tunnel excavation should be completed as soon as possible in weak grounds excavated under high overburden such as schists. Excavations and support systems should not be interrupted.

Possible face slidings on the slickenside surfaces in the schists unit are extremely important for tunnel stability. In these cases, excavation should be performed by soil nails to the tunnel face and an umbrella to the ceiling cut.

## Data Availability

All data generated or analysed during this study are included in this published article.
